# Boltz-2: Towards Accurate and Efficient Binding Affinity Prediction

**DOI:** 10.1101/2025.06.14.659707

**Published:** 2025-06-18

**Authors:** Saro Passaro, Gabriele Corso, Jeremy Wohlwend, Mateo Reveiz, Stephan Thaler, Vignesh Ram Somnath, Noah Getz, Tally Portnoi, Julien Roy, Hannes Stark, David Kwabi-Addo, Dominique Beaini, Tommi Jaakkola, Regina Barzilay

**Affiliations:** 1 MIT CSAIL; 2 MIT Jameel Clinic; 3 Valence Labs; 4 Recursion; 5 ETH Zurich

## Abstract

Accurately modeling biomolecular interactions is a central challenge in modern biology. While recent advances, such as AlphaFold3 and Boltz-1, have substantially improved our ability to predict biomolecular complex structures, these models still fall short in predicting binding affinity, a critical property underlying molecular function and therapeutic efficacy. Here, we present Boltz-2, a new structural biology foundation model that exhibits strong performance for both structure and affinity prediction. Boltz-2 introduces controllability features including experimental method conditioning, distance constraints, and multi-chain template integration for structure prediction, and is, to our knowledge, the first AI model to approach the performance of free-energy perturbation (FEP) methods in estimating small molecule–protein binding affinity. Crucially, it achieves strong correlation with experimental readouts on many benchmarks, while being at least 1000× more computationally efficient than FEP. By coupling Boltz-2 with a generative model for small molecules, we demonstrate an effective workflow to find diverse, synthesizable, high-affinity binders, as estimated by absolute FEP simulations on the TYK2 target. To foster broad adoption and further innovation at the intersection of machine learning and biology, we are releasing Boltz-2 weights, inference, and training code ^1^ under a permissive open license, providing a robust and extensible foundation for both academic and industrial research.

## 1 Introduction

Complex biological processes are governed by interactions between biomolecules, including proteins, DNA, RNA, and small molecules. In this work, we introduce Boltz-2, a new foundation model for elucidating biomolecular interactions. Building on its predecessors AlphaFold3 [Abramson et al., 2024] and Boltz-1 [Wohlwend et al., 2025], Boltz-2 improves structural accuracy across modalities, extends predictions from static complexes to dynamic ensembles and sets a new standard in physical grounding. However, its key distinctive feature is its ability to predict binding affinity, which measures how tightly small molecules attach to proteins. This measure is critical for understanding whether a drug will act on its intended target and be potent enough to produce a therapeutic effect.

Despite its importance in drug design, in-silico affinity prediction remains an open challenge. To date, the most accurate techniques are atomistic simulations like free-energy perturbations (FEP). However, they are far too slow and expensive to be used at scale. Faster methods, such as docking, are not precise enough to give a reliable signal. In fact, no AI-based model has yet matched the accuracy of FEP methods or laboratory assays for binding affinity prediction.

Boltz-2 overcomes this long-standing performance/compute time trade-off. This advancement builds on two complementary developments: data curation and representation learning. Finding the right training signal for this task is a known barrier. While large amounts of binding data are publicly available, in their raw form they are not suitable for training due to experimental differences and noise. To this end, we standardized millions of biochemical assay measurements, tailoring data curation, sampling and supervision to extract the useful signal from the data.

In terms of representation learning, affinity prediction builds on the latent representation driving the cofolding process. This representation inherently encodes rich information about biomolecular interactions. Therefore, Boltz-2’s improvements in binding affinity prediction are driven by advances in structural modeling. These stem from: (1) extending training data beyond static structures to include experimental and molecular dynamics ensembles; (2) significantly expanding distillation datasets across diverse modalities; and (3) enhancing user control through conditioning on experimental methods, user-defined distance constraints, and multi-chain template integration.

**Figure 1:**
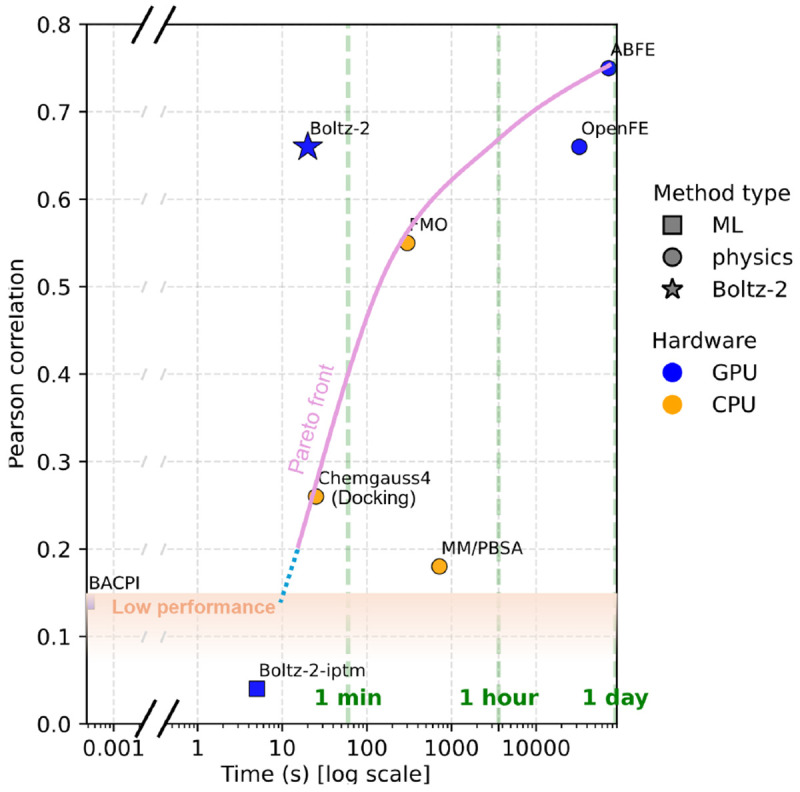
Boltz-2 presents a strong accuracy / speed trade-off for affinity prediction. Plot based on a 4-target subset (CDK2, TYK2, JNK1, P38) of the protein-ligand-benchmark [Hahn et al., 2022] for which baseline data are available for all methods. Full results in Figure 6.

The power of Boltz-2 to accurately predict affinity is evident in multiple discovery contexts:

- Hit-to-lead and lead optimization Boltz-2 significantly outperforms deep learning baselines on the FEP+ benchmark [Ross et al., 2023] and approaches the accuracy of FEP-based methods, while being over 1000 times faster (see Figure 1). On the CASP16 affinity track, retrospective evaluation shows that Boltz-2 outperforms all submitted competition entries out of the box.
- Hit discovery The model discriminates binders from decoys in high-throughput screens and achieves substantial enrichment gains on the MF-PCBA benchmark [Buterez et al., 2023], outperforming both docking and machine learning (ML) approaches.
- De-novo Generation Coupled with a generative model [Cretu et al., 2024], Boltz-2 enables discovery of new binders. In a prospective screening against the TYK2 target, this pipeline is able to generate diverse, synthetizable, high-affinity binders, as estimated by absolute binding free energy (ABFE) simulations [Wu et al., 2025].

Compared to Boltz-1, Boltz-2 improves crystallographic structure prediction across modalities, with notable gains on challenging targets such as antibody–antigen complexes. When benchmarked against molecular dynamics simulations, Boltz-2 matches the performance of recent specialized models, such as AlphaFlow [Jing et al., 2024] and BioEmu [Lewis et al., 2025], in predicting key dynamic properties like Root Mean Square Fluctuation (RMSF).

Alongside this manuscript, we are releasing Boltz-2’s model weights, inference pipeline and training code under a permissive open license. By making Boltz-2 freely available, we aim to accelerate progress across both academic and industrial efforts on tackling complex diseases and designing novel biomolecules. We also hope Boltz-2 will serve as a robust and extensible foundation for the growing machine learning community working at the interface of computation and biology, catalyzing further innovation in structure prediction, molecular design, and beyond.

## 2 Data

Aggregating and curating data are two of the most important steps in training strong foundational models. In this section, we summarize the training datasets and the key decisions made during data collection and preprocessing. Additional details are provided in Appendix A.

### Structural Data

For the structure model, we increased the diversity of biomolecules and data sources compared to Boltz-1. Unlike Boltz-1, which trained on a single structure per system, we supervise Boltz-2 using ensembles coming from both experimental techniques, such as NMR, as well as computational ones, such as molecular dynamics. The experimental data used for training comprises structures in the Protein Data Bank (PDB) [Berman et al., 2000] released before 2023–06-01. For molecular dynamics, we collected poses from the trajectories released as part of three large-scale open efforts: MISATO [Siebenmorgen et al., 2024], ATLAS [VanderMeersche et al., 2024], and mdCATH [Mirarchi et al., 2024]. Our goal is to expose Boltz-2 not only to single equilibrium points from crystal structures but also to local fluctuations and global structural ensembles.

To further improve the model’s understanding of local dynamics, we supervise the model’s single representation at the end of the trunk of the architecture to predict B-factors coming from both experimental methods as well as molecular dynamics trajectories.

In addition, we employ distillation to increase the size and diversity of the training data and its supervision signal. Distillation obtains additional training data by using high-confidence outputs of other models to augment the original training set. Specifically, we use AlphaFold2 high-confidence predictions on single-chain monomers [Varadi et al., 2022], like many previous models. Additionally, we employ high-confidence Boltz-1 prediction across a wide variety of complexes of single-chain RNA, protein-DNA, ligand-protein, MHC-peptide, and MHC-peptide-TCR interactions.

### Binding Affinity Data

Millions of binding affinity data points have been publicly released on central databases, such as PubChem [Kim et al., 2023] or ChEMBL [Zdrazil et al., 2024]; however, they have been notoriously difficult to combine into a single dataset for training due to variations in protocols and experimental noise [Landrum and Riniker, 2024].

Our data curation strategy focuses on: (1) retaining only the higher-quality assays, (2) mitigating overfitting to data biases by, for example, generating synthetic decoys, (3) ensuring structural quality by filtering targets with low confidence score, and (4) applying PAINS (pan-assay interference compounds) filters [Baell and Holloway, 2010] and discarding ligands with more than 50 heavy atoms.

Binding affinity predictions support two distinct tasks: hit discovery, where the goal is to identify likely binders across large chemical libraries, and hit-to-lead or lead optimization, where fine-grained affinity differences guide compound refinement. These use cases place different demands on the data: the former demands large-scale binary labeled data that distinguishes actives from inactives, while the latter requires precise, quantitative affinity measurements to resolve subtle activity differences. To support both settings, we curate a hybrid dataset comprising both binary and continuous labels. A summary of the resulting data is shown in Table 1.

For the binding affinity regression values (e.g., $K_{i}$, $K_{d}$, $IC_{50}$, $AC_{50}$, $EC_{50}$, $XC_{50}$), we gather data from PubChem [Kim et al., 2023], ChEMBL [Zdrazil et al., 2024], and BindingDB [Liu et al., 2007]. We retain only assays that target a single protein and are categorized as either biochemical or functional, excluding any labeled as low-confidence or unreliable. All affinity values are standardized to log_10_ scale derived from values measured in *μ*M. Assays with insufficient data or a low affinity standard deviation are discarded to encourage learning of intra-assay differences in values rather than inter-assay.

For the binary affinity classification data, we gather data from PubChem HTS (high-throughput screening) assays [Kim et al., 2023], a fragment screening dataset from CeMM [Offensperger et al., 2024], and MIDAS, a protein–metabolite interactome dataset from the University of Utah [Hicks et al., 2023]. For PubChem HTS, we retain only assays that include at least 100 compounds and exhibit a hit rate below 10%, helping to filter out noisy screens. To reduce false-positive labels introduced by HTS noise, we check for the presence of an associated quantitative affinity measurement (e.g., $K_{i}$, $K_{d}$, or $XC_{50}$) in independent assays. Lastly, we augment the binary classification dataset by generating synthetic decoys created by shuffling binders identified in hit-to-lead screens across different targets, while mitigating low false negative rates by ensuring that each decoy has a Tanimoto similarity below 0.3 to all known binders associated with similar proteins. This expands the pool of negative examples, improves coverage of the chemical space surrounding each protein target, and helps mitigate spurious correlations present in HTS assays.

## 3 Architecture

**Figure 2:**
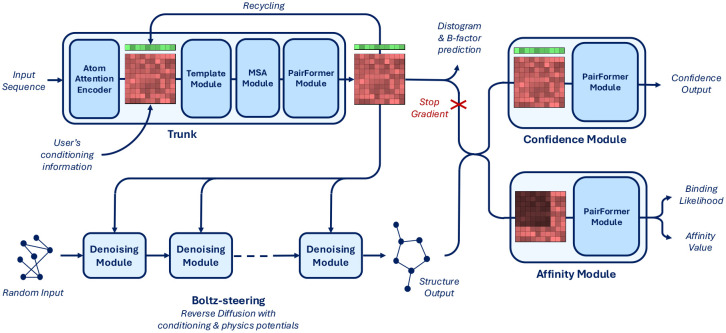
Boltz-2 model architecture diagram.

As shown in Figure 2, Boltz-2’s architecture comprises four main components: the trunk, the denoising module with additional steering components, the confidence module, and the affinity module. Below, we highlight the major differences compared to the Boltz-1 and Boltz-1x architectures, mostly related to the controllability components and the affinity module. Appendix B provides a detailed description of each component.

### Trunk optimization

The trunk is the most resource-intensive component of the model, largely due to the pairwise stack and triangular operations. We significantly improve the training and inference runtime as well as its memory consumption by using mixed-precision (bfloat16) and the trifast kernel for triangle attention. This also allows us to scale the crop size during training to 768 tokens, as done by AlphaFold3.

### Physical quality

Co-folding models such as AlphaFold3, Chai-1, and Boltz-1 often produce structures with physical inaccuracies such as steric clashes and incorrect stereochemistry [Abramson et al., 2024, Buttenschoen et al., 2024]. To address this, we recently introduced Boltz-steering (as part of the Boltz-1x release) — an inference-time method that applies physics-based potentials, which improves physical plausibility without sacrificing accuracy. We also integrate this approach within Boltz-2 to obtain Boltz-2x.

### Controllability

A frequent request from Boltz-1 users was a desire for more precise control of the model’s predictions, allowing them to test hypotheses or incorporate prior knowledge into the model without costly retraining or fine-tuning. To enable better controllability of the poses, we integrate three new components in Boltz-2: method conditioning, template conditioning and steering, and contact and pocket conditioning. Method conditioning allows for specification of the type of structure prediction method (e.g., X-ray crystallography, NMR, or molecular dynamics) that the predictions should align with and can capture their many nuances (see Section 5.2). Template conditioning integrates structures of similar complexes, helping the model without retraining [Jumper et al., 2021]. Unlike previous approaches, we allow users to either enforce strict observance of the templates via steering or just use the soft-conditioning like previous methods. As a departure from previous work, our templating approach also natively supports the use of multimeric templates. Finally, contact and p ocket conditioning allow for specification of particular distance constraints, whether they come from experimental techniques or human intuition.

### Affinity module

The affinity module consists of a PairFormer and two heads: one predicting binding likelihood, the other regressing continuous affinity values. During training, we supervise the affinity value head using a mixture of related, but non-identical biochemical quantities (including $K_{i}$, $K_{d}$, and $IC_{50}$) all converted to the logarithmic scale using *μ*M as standardized unit. While some of these measures are related through the Cheng–Prusoff equation, they arise from different experimental contexts. As such, the predicted value should be viewed as a general measure of binding strength that supports ranking and can be approximately interpreted as an $IC_{50}$-like value. The module operates on Boltz-2’s structural predictions, leveraging the pair representation and the predicted coordinates refined by a PairFormer model focused exclusively on the protein–ligand and intra-ligand interactions. These interactions are then aggregated to produce both a binding likelihood and an affinity value.

## 4 Training

The training of the model can be divided into three phases: structure training, confidence training, and affinity training. We further discuss how we use Boltz-2 to train a generative model for efficient exploration of the synthesizable chemical space. Full details on these components can be found in Appendix C.

### Structure and Confidence training

The structure and confidence training largely follows Boltz-1, with a few exceptions. (1) Computational optimizations allowed us to train the model for more iterations and larger crops. (2) Ensembles from experimental methods and molecular dynamics were supervised with an aggregated distogram to reduce variance. (3) The trunk’s final representation was also supervised to predict the B-factor of each token.

### Affinity training

Affinity training is performed after structure and confidence training, with gradients detached from the trunk. The pipeline incorporates several key components designed to improve generalization and scalability: pre-computation and cropping of binding pockets to focus on the most relevant interactions, pre-processing of trunk representations and a custom sampling strategy that balances binders and decoys while prioritizing informative, high-contrast assays. Batches are constructed to focus on local chemical variation. Supervision is applied jointly across binary and continuous affinity tasks using robust loss functions designed to mitigate the effects of experimental noise and assay heterogeneity. Continuous values are supervised using a Huber loss applied to both absolute affinity values and, with stronger weight, to the pairwise intra-assay differences. We observed best performance when training a single affinity value head on all available affinity measurements (eg, $K_{i}$, $K_{d}$, $IC_{50}$, $AC_{50}$, $EC_{50}$, and $XC_{50}$). Although these metrics reflect different underlying biochemical quantities, $K_{i}s$ and $IC_{50}s$ are related through the Cheng–Prusoff equation, and when comparing affinity values within the same assay, the pairwise differences loss effectively cancel out the correction term, and assays can be combinedRoss et al. [2023]. Binary classification is supervised using a focal loss [Lin et al., 2017] to address class imbalance and reduce overfitting. The final training objective is a weighted combination of the classification and the regression losses, designed to balance the different tasks.

### Training a molecular generator with Boltz-2

As part of our evaluation, Boltz-2 is used to train a molecular generator to produce small molecules with high binding scores. Our generative agent (SynFlowNet [Cretu et al., 2024]) employs a GFlowNet [Bengio et al., 2021] loss function, enabling it to sample from arbitrary and multi-modal score distributions. Within this framework, the molecular generator undergoes off-policy training: batches of candidate molecules are asynchronously submitted to Boltz-2 workers for scoring, and the results are then incorporated into a replay buffer for the generative agent. The binding score (reward) for the agent is a strictly positive metric derived from a combination of both the binding likelihood and affinity values predicted by Boltz-2. The training procedure also incorporates basic drug-likeness properties through medicinal chemistry filters.

## 5 Evaluation

In this section, we evaluate Boltz-2 in various settings, including crystal structure prediction, local protein dynamics, binding likelihood and affinity predictions, and virtual screening. For the affinity measurements, all cross-assay averages are weighted by the number of compounds in the assay and error bars are computed as the bootstrapping standard deviation.

### 5.1 Boltz-2 improves over Boltz-1 on structure prediction

#### PDB evaluation set.

**Figure 3:**
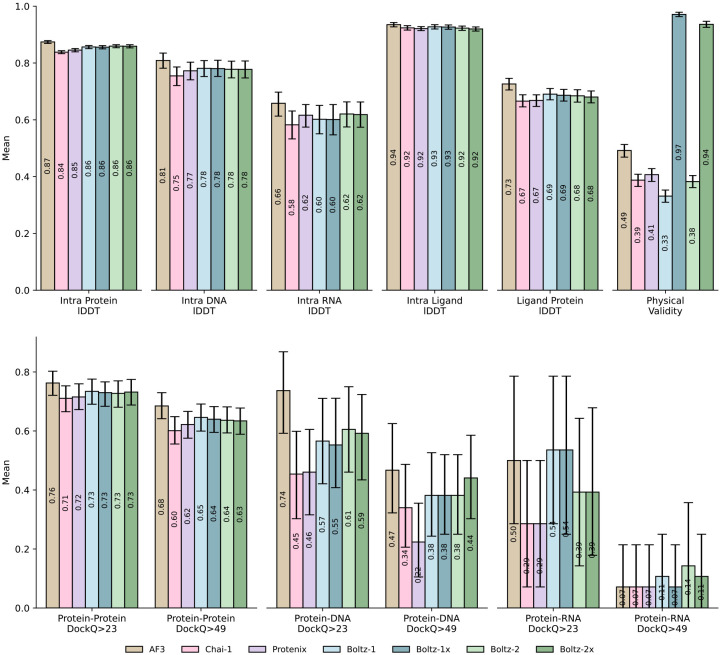
Evaluation of the performance of Boltz-2 against existing co-folding models on a diverse set of unseen complexes. Error bars indicate 95% confidence intervals.

We evaluated the performance of Boltz-2 (and its version with enabled physicality steering potentials, Boltz-2x), comparing it against Boltz-1, Chai-1 [Chai et al., 2024], ProteinX [Chen et al., 2025], and AlphaFold3 and across a wide variety of complexes submitted to the Protein Data Bank in 2024 and 2025 that were significantly different from any structure that any of the models had seen in their training set. The results, presented in Figure 3, show that, across modalities, Boltz-2 matches or moderately improves over the performance of Boltz-1. Among the modalities where the improvements are strongest are RNA chains and DNA-protein complexes. These are the two modalities where we most significantly augmented the available data in the PDB with large distillation sets, suggesting that the distillation strategy could be important to improve these models beyond what available experimental data allows. Compared also to other methods, Boltz-2 performs competitively, edging the other commercially available models Chai-1 and ProteinX, but lagging a bit behind AlphaFold3. As expected Boltz-1x and Boltz-2x, thanks to Boltz-steering obtain significantly better physicality metrics both for small-molecule conformations and for steric clashes at interfaces.

#### Antibody benchmark.

**Figure 4:**
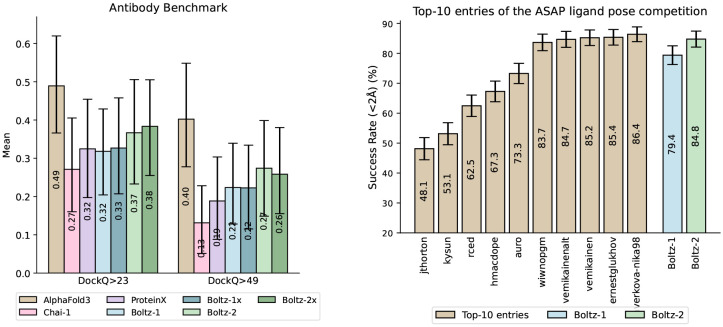
*Left:* Performance of different co-folding methods on a challenging antibody benchmark. Boltz-2 shows an improvement over Boltz-1 while still lagging behind AlphaFold3. *Right:* Retrospective results for the Polaris-ASAP competition, with Boltz-2 matching the performance of the top 5 contenders without any fine-tuning or physics relaxation. Error bars indicate 95% confidence intervals.

One modality where researchers have highlighted a performance gap between AlphaFold3 and the commercially-available models is antibody-antigen structure prediction, especially when looking at the generalization to unseen antigens. This observation is also reflected in the results from our antibody benchmark shown in Figure 4. However, we also observe a moderate improvement of Boltz-2 over Boltz-1, narrowing the gap between the proposed open models and proprietary ones, such as AlphaFold3.

#### Polaris-ASAP challenge (SARS-CoV-2 and MERS-CoV)

We further evaluated the model on the recent Polaris-ASAP Discovery competition on ligand pose estimation. This was composed of ligands bound to either the SARS-CoV-2 and MERS-CoV main proteases that ASAP Discovery generated as part of their antiviral drug discovery campaigns. On top of the PDB, 770 additional structures of similar ligands bound to these proteins were given as a training set. This challenge saw a clear success of co-folding models over more traditional physics-based and ML tools, with all the top-6 entries being composed of fine-tuned Boltz-1 or AlphaFold3 models (some with additional physics-based relaxation). Boltz-2 shows a clear improvement over Boltz-1 and the top performers in the challenge, without any finetuning or physics-based relaxation (Figure 4 right).

### 5.2 Boltz-2 can better capture local protein dynamics

**Figure 5:**
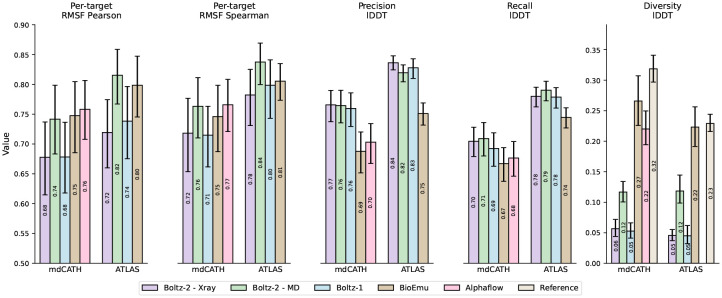
Per-target RMSF correlation metrics and lDDT metrics on the held-out clusters from the mdCATH and ATLAS molecular dynamics datasets.

In order to validate the impact of MD method conditioning and evaluate the model’s ability to capture local dynamics of protein structures, we evaluated Boltz-2 on the held-out clusters of the mdCATH and ATLAS datasets. The results, presented in Figure 5 and Appendix E.1, show that (1) MD conditioning has a clear effect on the predicted ensembles, leading to more diverse structures that better capture the conformational diversity of the simulations, (2) Boltz-2 with MD conditioning is competitive on various metrics with specialized models such as BioEmu [Lewis et al., 2025] and AlphaFlow [Jing et al., 2024]. When looking at RMSF, a standard measure of local dynamics, Boltz-2 MD ensembles generally obtain stronger correlations with the ground truth simulation and lower errors than Boltz-1, BioEmu and AlphaFlow. In addition to training on MD ensembles, Boltz-2’s performance may also benefit from supervision on both experimental and computational B-factor estimates, which are specifically designed to capture local structural dynamics. Looking at recall lDDT, Boltz-2 modestly outperforms Boltz-1 while improving over AlphaFlow and BioEmu. Conditioning on MD allows Boltz-2 to increase the diversity of samples while retaining its precision. This diversity increase is, however, outperformed by BioEmu and AlphaFlow, which more closely align with the reference diversity from the simulation.

### 5.3 Boltz-2 approaches FEP accuracy on public benchmarks

Accurately ranking analogues within a chemical series is a critical challenge in hit-to-lead and lead optimization. Distinguishing subtle differences in binding affinity among closely related analogues is essential for guiding molecular refinement and progressing candidates through the pipeline. Traditional free energy simulation methods can often offer the required precision, but are too computationally expensive for more widespread use. Boltz-2 addresses this problem as it allows accurate affinity predictions at a fraction of the computational cost, enabling rapid prioritization in structure-guided optimization workflows.

**Figure 6:**
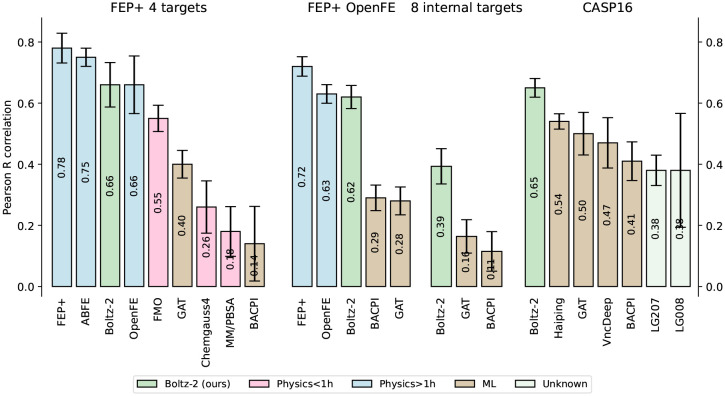
Pearson correlation averaged over each assay on our four affinity value test sets. Error bars represent bootstrap estimates of the standard error.

To evaluate Boltz-2’s affinity prediction ability, we benchmarked it across a suite of hit-to-lead and lead-optimization datasets. Summary results are presented in Figure 6, while expanded tables and scatter plots are available in Appendices E.2.1 and E.2.2.

We evaluate the model on two subsets of the FEP+ benchmark [Ross et al., 2023]: the OpenFE dataset, consisting of 876 high-quality hit-to-lead measurements [Gowers et al., 2023], and a focused 4-target subset [Hahn et al., 2022], where more physics-based baselines are available, including absolute FEP (ABFE) [Wu et al., 2025] and Fragment Molecular Orbital (FMO) [Nishimoto and Fedorov, 2016, Guareschi et al., 2023], a semi-empirical quantum mechanics-based scoring function. The training sets are filtered to exclude proteins with ≥ 90% sequence identity to any protein in the FEP+ benchmark, ensuring that we benchmark on unseen proteins. Additionally, we assess the impact of compound similarity in Figure D.2.1. On the 4-target FEP subset, Boltz-2 achieves an average Pearson correlation of 0.66, outperforming all available inexpensive physical methods and ML baselines. Remarkably, Boltz-2 approaches state-of-the-art free energy simulations, while running more than 1,000× faster, providing a strong speed-accuracy tradeoff (Figure 1). Even on the full OpenFE benchmark set, Boltz-2 approaches the performance of OpenFE, a widely adopted open-source relative FEP method.

Additionally, we include the CASP16 affinity challenge [Gilson et al., 2025], a rigorous blind benchmark featuring 140 protein–ligand pairs across two targets. Here, while participants were given several weeks and used a range of ad-hoc machine learning and physics-based tools, we ran Boltz-2 out-of-the-box with no fine-tuning or input curation. Yet, Boltz-2 outperforms all top-ranking participants by a clear margin.

We also evaluated the model on eight blinded internal assays from Recursion that reflect complex real-world medicinal chemistry projects. Here, the model still outperforms by a large margin the other ML baselines and achieves a Pearson correlation of > 0.55 on 3 out of 8 assays, but has limited performance on the other 5. Such variation is also typical of FEP methods, which are known to perform weakly on some protein classes, such as GPCRs, without custom input preparation [Deflorian et al., 2020]. We include these results as a reminder that strong performance on public benchmarks does not always immediately translate to all complexities of real-world drug discovery without further work to understand the relative strengths and weaknesses of a given approach.

### 5.4 Boltz-2 enables accurate large-scale virtual screening

Accurate virtual screening remains one of the most impactful challenges in early-stage drug discovery. The ideal method must scale across vast chemical libraries while reliably identifying active compounds against diverse protein targets. Boltz-2 offers a promising solution to this problem, combining speed and precision in a unified affinity prediction framework.

**Figure 7:**
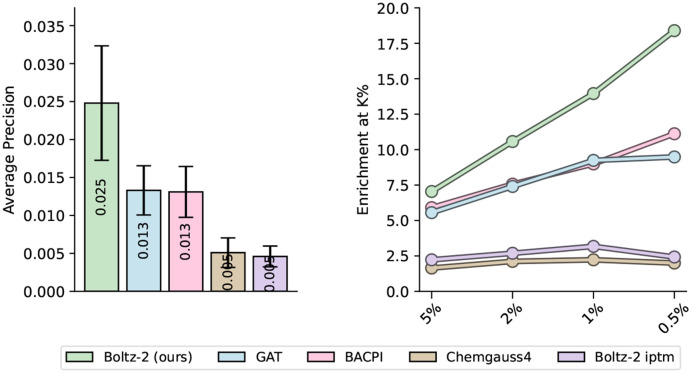
*Left:* Average precision averaged over the assays in the MF-PCBA test set. Error bars represent bootstrap estimates of the standard error. *Right:* Enrichment factors, computed at top-K thresholds with K = 0.5%, 1%, 2%, and 5%.

To assess its utility in realistic screening settings, we first evaluated Boltz-2 on retrospective benchmarks derived from the MF-PCBA dataset [Buterez et al., 2023], which includes high-quality biochemical assays spanning diverse protein families. Performance was assessed using metrics tailored to hit discovery—average precision (AP), enrichment factor at top-ranked percentiles, and AUROC. Results highlight Boltz-2’s ability to retrieve actives from large, imbalanced datasets (Figure 7). On this benchmark, Boltz-2 substantially outperforms prior machine learning approaches, the widely used ipTM and docking, nearly doubling the average precision and achieving an enrichment factor of 18.4 at a 0.5% threshold (Table 13).

To evaluate Boltz-2 in prospective settings, we performed a virtual screen against the kinase target TYK2, a protein well-characterized in both ML and physics-based modeling benchmarks. We selected TYK2 for two main reasons: First, TYK2 is in the test set of the Boltz-2 affinity model, avoiding data leakage from known binders. Second, in the absence of experimental data, we validate the compounds selected by Boltz-2 with a single repeat of Boltz-ABFE^2^ [Wu et al., 2025], our recently developed absolute FEP pipeline to estimate ABFE values without experimental crystal structures, and Boltz-ABFE performs very well on this target. Indeed, based on the protein-ligand benchmark [Hahn et al., 2022], Boltz-ABFE achieves a Pearson R = 0.95, centered MAE = 0.42 kcal/mol and a comparatively small offset with respect to the experiment of 0.92 kcal/mol, supporting our confidence of this procedure as a validation step for TYK2-targeting virtual screens.

**Figure 8:**
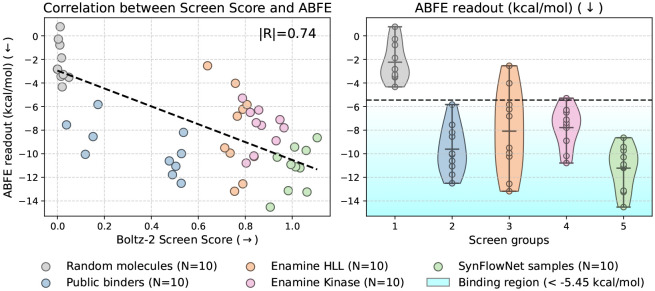
Virtual screening experiment performed on the TYK2 protein. *Left:* The Boltz-2 screen scores of the final set of compounds of each virtual screening stream correlate (∣R∣=0.74) with the absolute binding free energy (ABFE) estimates ΔG. *Right:* Distribution of the ABFE-predicted ΔG for the compounds proposed by the different screening strategies.

In these screens, we use a combination of the Boltz-2 predicted binding likelihood and affinity as a screen score for small molecules. We started by screening two commercially available compound libraries from Enamine—Hit Locator Library (HLL, 460,160 compounds) and Kinase Library (64,960 compounds). Boltz-2 successfully prioritized high-affinity ligands: Based on ABFE estimates, 8 of the top 10 compounds from HLL and all 10 compounds from the Kinase library are predicted to bind, while all 10 random compounds are predicted to be non-binders (Figure 8).

We further extended this screening pipeline using a generative approach. Boltz-2 was coupled with SynFlowNet [Cretu et al., 2024], a GFlowNet-based molecular generator designed to sample molecules from Enamine’s 76B REAL space (details in Appendices B.6 and C.3). This generative screen offers a scalable alternative to fixed libraries by exploring synthesizable chemical space beyond off-the-shelf compounds. After scoring, filtering, and diversity selection, we submitted 10 de novo candidates for ABFE simulation (see Appendix E.3). All selected compounds from the SynFlowNet stream are predicted to bind TYK2, with higher affinity on average compared to fixed screens, and while requiring substantially less computational budget than the HLL screen (117k Boltz-2 evaluations for SynFlowNet against 460k evaluations for HLL). Visualisations of all the selected compounds and of the top-2 ABFE-scored ligand-protein complexes for each stream are presented in Appendix E.3.3. Finally, in Appendix E.3.4, we further assess the novelty of the SynFlowNet-generated compounds by examining their Tanimoto similarity with known binders from the PDB that are part of the structure module training data, and find that the generated compounds do not exhibit significant similarity to public TYK2 binders. We note that these results might be optimistic given that Boltz-2 performs well on this target based on the protein-ligand benchmark data [Hahn et al., 2022], achieving Pearson R= 0.83.

Together, these results demonstrate how Boltz-2 enables structure-based prioritization at a large scale. By addressing both performance and scalability, Boltz-2 expands the scope of target-based in-silico optimization to large scale, encompassing hit discovery, hit-to-lead, and lead optimization.

## 6 Limitations

Despite the progress made in this work for structure and binding affinity prediction, we acknowledge several remaining limitations of the model that we aim to address in future work.

### Molecular Dynamics

While there are clear improvements over Boltz-1, the model does not significantly deviate from other baselines such as AlphaFlow or BioEmu. The current model used a relatively small MD dataset at the later stages of training, with minor architectural changes to account for multiple conformations. Additional changes from the modeling perspective, as well as the datasets used, are required to further improve its capabilities.

### Remaining challenges for structure prediction.

While we see a consistent improvement in the structure prediction performance from Boltz-1 to Boltz-2, the model does not significantly deviate from the structure prediction performance of its predecessors. This similarity is primarily due to the use of largely identical structural training data, a similar architectural design, and withstanding limitations in predicting complex interactions, particularly within large complexes. In addition, the model still often fails to capture large conformational changes, such as those that can be induced by binding.

### Accurate structures for affinity predictions.

Boltz-2 relies on predicted 3D protein–ligand structures and reliable trunk features as input to the affinity module. If the model fails to identify the correct p ocket or inaccurately reconstructs the binding interface or conformational state of the protein, downstream affinity predictions are unlikely to be reliable. This is particularly relevant in biological contexts where cofactors are essential for binding, given that in its current form, the affinity module does not explicitly handle such cofactors, including ions, water, or multimeric binding partners. Finally, an insufficiently large affinity crop size could be limiting if important long-range interactions are truncated or if the crop does not include the corresponding po cket for each binder, e.g., in the case of both orthosteric and allosteric modulators.

### Understanding the range of applicability of the affinity module.

Despite the progress on affinity predictions, we notice in Figures 12-14 that the performance varies strongly between assays. Further work is needed to determine the source of this variance in performance, whether it stems from, e.g., inaccuracies in predicted structures, limited generalization to distinct protein families, or insufficient robustness to out-of-distribution small molecules.

## 7 Conclusion

We introduce Boltz-2, a new structural biology foundation model that advances the frontiers of both structure and affinity prediction. Boltz-2 builds on the co-folding capabilities of its predecessor with improved physical plausibility, fine-grained controllability, and a better understanding of local dynamics. Our results show that Boltz-2 performs competitively across a broad range of structure prediction tasks, including challenging modalities and conformational ensembles derived from MD. Crucially, Boltz-2 is, to our knowledge, the first AI model to approach the accuracy of FEP methods for predicting binding affinities on the FEP+ benchmark, while offering orders-of-magnitude gains in computational efficiency. For affinity values, Boltz-2 demonstrates strong retrospective and prospective performance in both hit discovery, hit-to-lead and lead optimization settings, as observed on many assays across public benchmarks, private benchmarks, and virtual screening workflows. Coupled with a generative model for small molecules, Boltz-2 enables an end-to-end framework for de novo binder generation, which is validated through ABFE simulations on the TYK2 protein. Despite these advances, several limitations remain, as outlined above. Addressing these issues will require future work in expanding and curating training data, refining model architecture, and integrating additional biochemical context.

By releasing Boltz-2 and its training pipeline under a permissive license, we aim to support the growing community working at the intersection of AI and molecular science. We hope Boltz-2 will serve as a foundation for further advances in drug discovery, protein design, and synthetic biology, expanding the boundaries of what is computationally possible in biomolecular modeling.

## Supplementary Material

1

## Figures and Tables

**Table 1:** Summary statistics of the affinity training dataset used in our model. Each row corresponds to a different data source or curation strategy. The table reports the number of binders, decoys, unique protein clusters at 90% sequence identity (referred to as Targets in the table), and compounds. Supervision indicates whether the data is used to supervise the binary and/or affinity value head. Values in parentheses show the corresponding statistics prior to applying the structural quality filter, which excludes examples with iptm below 0.75.

Source	Type	Supervision	# Binders	# Decoys	# Targets	# Compounds
ChEMBL and BindingDB	optimization	values	1.2M (1.45M)	0	2k (2.5k)	600k (700k)
PubChem small assays	hit-discovery	both	10k (13k)	50k (70k)	250 (300)	20k (25k)
PubChem HTS	hit-discovery	binary	200k (400k)	1.8M (3.5M)	300 (500)	400k (450k)
CeMM Fragments	hit-discovery	binary	25k (45k)	115k (200k)	1.3k (2.5k)	400 (400)
MIDAS Metabolites	hit-discovery	binary	2k (3.5k)	20k (35k)	60 (100)	400 (400)
ChEMBL and BindingDB	synthetic decoys	binary	0	1.2M (1.45M)	2k (2.5k)	600k (700k)

## Footnotes

2 Preprint available soon.
