# Supplementary material for: Boltz-2: Towards Accurate and Efficient Binding Affinity Prediction
Source: bioRxiv. 2025 Jun 18:2025.06.14.659707. Preprint. [Version 1] doi: 10.1101/2025.06.14.659707 (PMC12262699; doi:10.1101/2025.06.14.659707)
Supplement: 1 [file NIHPP2025.06.14.659707v1-supplement-1.pdf]

bioRxiv preprint doi: https://doi.org/10.1101/2025.06.14.659707; this version posted June 18, 2025. The copyright holder for this preprint (which was not certified by peer review) is the author/funder, who has granted bioRxiv a license to display the preprint in perpetuity. It is made available under aCC-BY 4.0 International license.

Supplementary Material

Contents

1 Introduction **1**

2 Data **3**

3 Architecture **4**

4 Training **5**

5 Evaluation **6**
5.1 Boltz-2 improves over Boltz-1 on structure prediction . . . 6
5.2 Boltz-2 can better capture local protein dynamics . . . 7
5.3 Boltz-2 approaches FEP accuracy on public benchmarks . . . 9
5.4 Boltz-2 enables accurate large-scale virtual screening . . . 10

6 Limitations **11**

7 Conclusion **12**

Supplementary Material **14**

A Data **16**
A.1 Structural Data . . . 16
A.1.1 PDB data . . . 16
A.1.2 Molecular Dynamics data . . . 16
A.1.3 Distillation data . . . 17
A.1.4 Training sampling weights . . . 18
A.1.5 Validation dataset . . . 18
A.2 Binding Affinity Data . . . 19
A.2.1 Affinity values . . . 20
A.2.2 Binary labels . . . 21

B Model **23**
B.1 Tokenization and Featurization . . . 23
B.2 Trunk and Denoising Modules Architecture . . . 23
B.3 Controllability . . . 24
B.3.1 Method conditioning . . . 24
B.3.2 Templates conditioning and steering . . . 24
B.3.3 Contacts and pocket conditioning and steering . . . 24
B.4 Confidence Module Architecture . . . 25
B.5 Affinity Module Architecture . . . 25
B.5.1 Affinity Module . . . 26
B.5.2 Affinity Model Ensemble . . . 26

bioRxiv preprint doi: https://doi.org/10.1101/2025.06.14.659707; this version posted June 18, 2025. The copyright holder for this preprint (which was not certified by peer review) is the author/funder, who has granted bioRxiv a license to display the preprint in perpetuity. It is made available under aCC-BY 4.0 International license.

B.6 SynFlowNet Ligand-Generation Model . . . 27

C Training **28**
C.1 Structure and confidence training . . . 28
C.1.1 Main differences with Boltz-1 . . . 28
C.1.2 Controllability Training . . . 28
C.1.3 Training stages . . . 29
C.1.4 Architecture, Training and Inference Hyperparameters . . . 29
C.2 Affinity training . . . 30
C.2.1 Pocket pre-processing . . . 30
C.2.2 Affinity cropper . . . 30
C.2.3 Feature pre-processing . . . 31
C.2.4 Training sampler . . . 31
C.2.5 Affinity supervision . . . 32
C.3 Generative ligand optimization with SynFlowNet . . . 33

D Benchmarks and Baselines **35**
D.1 Structure prediction . . . 35
D.1.1 Benchmarks sets curation . . . 35
D.1.2 Evaluation metrics . . . 35
D.1.3 Baselines . . . 35
D.2 Affinity prediction . . . 36
D.2.1 Benchmark sets curation . . . 36
D.2.2 Evaluation metrics . . . 37
D.2.3 Baselines . . . 38

E Extended Results **40**
E.1 Molecular Dynamics . . . 40
E.2 Affinity prediction . . . 42
E.2.1 Public benchmarks . . . 42
E.2.2 Private benchmarks . . . 43
E.3 Prospective virtual screens . . . 46
E.3.1 Ligand filtering pipeline . . . 46
E.3.2 ABFE validation protocol . . . 47
E.3.3 Ligands visualisations . . . 47
E.3.4 Similarity analysis to public TYK2-binders . . . 49

bioRxiv preprint doi: https://doi.org/10.1101/2025.06.14.659707; this version posted June 18, 2025. The copyright holder for this preprint (which was not certified by peer review) is the author/funder, who has granted bioRxiv a license to display the preprint in perpetuity. It is made available under aCC-BY 4.0 International license.

A Data

A.1 Structural Data

The training data for Boltz-2 can be divided into two categories, structural and affinity.

The data used for the structural training is an extension of the Boltz-1 data from the PDB [Berman et al., 2000] to include the extractions of ensembles and B-factors [Sun et al., 2019] for supervision and templates for training. Beyond the PDB, it further integrates datasets obtained from molecular dynamics simulations as well as distillation datasets generated with AlphaFold2 and Boltz-1 predictions.

A.1.1 PDB data

We process every structure in the PDB following a pipeline similar to those previously described in Boltz-1 [Wohlwend et al., 2025] and AlphaFold3 [Abramson et al., 2024]:

- We use every PDB structure up to the training date cutoff of 06/01/2023. We parse the Biological Assembly 1 from these structures.
- For each polymer chain, we use the reference sequence and align it to the residues available in the structure.
- For ligands, we refer to the CCD dictionary to get the reference ligand and atom composition. We compute up to 10 3D conformers per ligand and sample one at random during training.
- When multiple models are present in the file, such as for NMR structures, we individually process each frame, ensuring that the atomic composition is consistent across frames for the full complex.
- We remove large complexes that are over 7MB or with more than 5000 residues.
- We apply the same filters as AlphaFold3, namely excluding crystallization aids and other non-biologically relevant ligands, removing clashing chains, and filtering out chains with fewer than 4 resolved residues or composed only of unknown residues.
- We compute multiple-sequence alignments for every protein chain (and only protein chains) using ColabFold search. Once monomeric MSAs are produced, we assign a taxonomy ID to every sequence in every MSA using their Uniref100 IDs as reference, if any. The preprocessing of the MSAs is analogous to AlphaFold3.
- We produce template hits for protein chains as described in AlphaFold3, using hmmbuild and hmmsearch on PDB sequences deposited at least 60 days prior to any given query's deposition date.
- We extract, when available, the B-factors from PDB entries.

A.1.2 Molecular Dynamics data

Multiple publicly available molecular dynamics datasets were used for training:

1. *MISATO*: We downloaded the MD dataset from Siebenmorgen et al. [2024], containing NVT trajectories simulated at 300K for 8ns. Trajectories with multi-residue ligands (such as glycans) or modified peptides as ligands are discarded. Ligand matchings to CCD codes are performed by using the reference and mol2 files from PDBbind [Liu et al., 2015]. Trajectories where the ligand floats away from the protein for more than 12Å at any frame are removed. Structures in the dataset may contain chain breaks that differ from the corresponding original entries in the PDB. To match these fragments to the appropriate chains and entities in the PDB, their sequences are matched to both the reference author sequence and the structure-derived sequences in the biological assembly 1 with `Align.PairwiseAligner`. Entries with less than 90% similarity are discarded. Non-overlapping chains that match substrings of the original PDB sequences are

bioRxiv preprint doi: https://doi.org/10.1101/2025.06.14.659707; this version posted June 18, 2025. The copyright holder for this preprint (which was not certified by peer review) is the author/funder, who has granted bioRxiv a license to display the preprint in perpetuity. It is made available under aCC-BY 4.0 International license.

greedily merged when possible. Molecular types, entity information and MSA information are assigned after matching to the corresponding parsed PDB data. Polymer and ligands are then parsed with the same pipeline used to parse the PDB, as described above. All 100 frames from the 8ns are parsed and used for training. After all filters are applied, the dataset results in 11,235 systems.

2. *ATLAS*: We downloaded the MD dataset from Vander Meersche et al. [2024], containing NPT trajectories simulated at 300K for 100ns. Structures are matched to their corresponding PDB entries. Replica trajectories are aggregated, and 100 frames are uniformly sampled at random from the final 10 nanoseconds of each trajectory for training, resulting in a final dataset of 1,284 proteins.

3. *mdCATH*: Trajectories from Mirarchi et al. [2024] containing NVT trajectories at 320K with varying simulation times up to 500ns were used. For training, only the last 10% of the trajectory was utilized. The final dataset comprises 5,270 systems.

A.1.3 Distillation data

For training the Boltz-2 model, we used several datasets generated by distilling predictions from the Boltz-1 model, while applying appropriate filters. For all datasets generated from Boltz-1, we employed 3 recycling steps in the trunk and generated 3 diffusion samples per example.

1. *RNA distillation*: Following AlphaFold3, we clustered Rfam (v14.9) [Kalvari et al., 2021] using MMSeqs2 [Steinegger and Söding, 2017] with 90% identity and 80% coverage. To form the distillation set, Boltz-1 predictions for cluster representatives are filtered to those where the maximum average predicted distance error (PDE) ≤ 2.0.

2. *Protein-DNA distillation*: Our Protein-DNA distillation data is constructed similarly to AlphaFold3. Using the JASPAR 2024 release (specifically, the CORE collection), we first find transcription factor profiles with matching gene IDs across two high-throughput SELEX datasets [Jolma et al., 2015, Yin et al., 2017]. For each filtered profile, a protein sequence is assigned in two ways: i) using the canonical protein sequence under the profile's Uniprot ID and ii) searching for the sequence in the two SELEX datasets (with matching gene ID) with the highest similarity to the Uniprot sequence. Sequence similarity is calculated using KAlign v2.0, computed as the number of non-gap matches between the two sequences divided by the minimum length of pre-aligned sequences. Unlike AlphaFold3, we did not apply any sequence clustering. To generate binding DNA sequences for each protein sequence, we use the corresponding JASPAR profile's position frequency matrix (PFM) to sample 10 single-stranded motifs. For each distillation example, the inputs include the protein sequence, the single-strand DNA sequence and its corresponding reverse complement. After generating Boltz-1 predictions, we filtered examples to those that satisfied all the following conditions PDE ≤ 2.0, maximum interface predicted distance error (iPDE) ≤ 1.0 and minimum interface predicted TM-Score (ipTM) ≥ 0.7.

3. *RNA-Ligand distillation*: We use the R-SIM [Krishnan et al., 2023] dataset comprising ~ 2500 examples of RNA-small molecule affinities. Predictions from Boltz-1 for these examples are filtered to those with either iPDE ≤ 1.0 or (ipTM) ≥ 0.7.

4. *Protein-Ligand distillation*: We construct a dataset of protein-ligand distillation from BindingDB and ChEMBL that were excluded from the main hit-to-lead affinity training set due to having too few compounds in the assay. The full parsing and filtering criteria applied are described in Appendix A.2. The distillation set was formed by filtering Boltz-1 predictions to examples with a maximum interface predicted distance error (iPDE) ≤ 1.0 and a minimum interface predicted TM-Score (ipTM) ≥ 0.9.

5. *TCR-pMHC distillation*: We generate the TCR self-distillation dataset (class I and class II) from VDJdb [Goncharov et al., 2022]. We retain only paired TCR entries with complete gene annotations, enabling accurate reconstruction of full-length TCR sequences using Thimble, the bulk-processing version of Stitchr [Heather et al., 2022]. Entries flagged with reconstruction

bioRxiv preprint doi: https://doi.org/10.1101/2025.06.14.659707; this version posted June 18, 2025. The copyright holder for this preprint (which was not certified by peer review) is the author/funder, who has granted bioRxiv a license to display the preprint in perpetuity. It is made available under aCC-BY 4.0 International license.

errors by Thimble are removed. The inputs for Boltz-1 contained TCR alpha and beta sequences (cropped to 9 residues beyond the CDR3 region), peptide sequences, and MHC alpha/beta sequences (cropped at 180 residues). Multiple Sequence Alignments (MSAs) are generated using ColabFold [Mirdita et al., 2022] and included for TCR and MHC chains, but not peptides. The distillation set was formed by filtering Boltz-1 predictions to examples with a (iPDE) ≤ 1.0, PDE ≤ 1.0, and (ipTM) ≥ 0.8.

6. *MHC-I and MHC-II*: We generate the pMHC self-distillation dataset (class I and class II) from the Immune Epitope Database (IEDB) [Vita et al., 2025]. We initially filter for epitopes with clearly annotated MHC alleles, remove mutant epitopes, and exclude sequences already present in the Protein Data Bank (PDB). Class I epitopes were restricted to 8-12 residues and class II epitopes to 13-25 residues. For class 1, we included MHCs from across multiple species. For class 2 MHCs, we only use human MHC alleles and discard entries for which we could not determine standardized and paired MHC sequences. For each MHC allele, we crop sequences to a maximum length of 180 residues. To limit redundancy, we sample up to 100 sequences per allele for class I and up to 200 per allele pair for class II. The distillation set is formed by filtering Boltz-1 predictions to examples with a (iPDE) ≤ 1.0, PDE ≤ 1.0, and (ipTM) ≥ 0.85.

7. *AlphaFold Database (AFDB) distillation:* In order to construct a protein monomer distillation set, we begin with uniref30 and find the overlap between those sequences and the uniclust multiple sequence alignments provided by OpenFold. We then fetch structures from the AFDB where we impose a minimum global lDDT of 0.5. This procedure results in a monomer distillation of about 5 million proteins.

A.1.4 Training sampling weights

Table 2 summarizes the various datasets used for the structure training and details their relative sampling weights. As shown, from the PDB we upsample interfaces containing antibodies and TCR, as these are specific modalities that we wanted the model to improve on, as well as proteins similar to SARS-CoV2-Mpro as we had planned to participate in the Polaris-ASAP competition.

Dataset	Source	Sampling Clusters	Sampling weight
PDB	experimental	chains & interfaces	0.455
Antibodies upsampling	experimental	interfaces	0.025
TCR upsampling	experimental	interfaces	0.004
SARS-Cov2-Mpro upsampling	experimental	interfaces	0.015
AFDB	AF2 distillation	chains	0.380
Protein-ligand	Boltz-1 distillation	interfaces	0.031
RNA	Boltz-1 distillation	chains	0.045
DNA-protein	Boltz-1 distillation	interfaces	0.020
RNA-ligand	Boltz-1 distillation	interfaces	0.001
TCR	Boltz-1 distillation	interfaces	0.012
pMHC-I	Boltz-1 distillation	interfaces	0.006
pMHC-II	Boltz-1 distillation	interfaces	0.006
ATLAS	MD	chains	0.003
MISATO	MD	chains & interfaces	0.230
mdCATH	MD	chains	0.010

Table 2: Breakdown of training data sources and their relative sampling weights. Sampling clusters indicate how elements are counted in the sampler that selects the complexes to train on and also determines where the first token of the crop is placed.

A.1.5 Validation dataset

PDB data. Our training, validation and test splitting strategy largely follows Boltz-1 procedure [Wohlwend et al., 2025]. We first cluster the protein sequences in PDB by sequence identity with the

bioRxiv preprint doi: https://doi.org/10.1101/2025.06.14.659707; this version posted June 18, 2025. The copyright holder for this preprint (which was not certified by peer review) is the author/funder, who has granted bioRxiv a license to display the preprint in perpetuity. It is made available under aCC-BY 4.0 International license.

command mmseqs easy-cluster ... –min-seq-id 0.4 [Hauser et al., 2016]. Then, we select all structures in PDB satisfying the following filters:

1. Initial release date is before 2023-06-01 (exclusive) and 2024-01-01 (inclusive).
2. Resolution is below 4.5 Å.
3. All the protein sequences of the chains are not present in any training set clusters (i.e. before 2023-06-01).
4. Either:
   - No small-molecule is present.
   - At least one of the small-molecules exhibits a Tanimoto similarity of 0.85 or less to any small-molecule in the training set. Here, a small-molecule is defined as any non-polymer entity containing more than one heavy atom and not included in the ligand exclusion list.
   - The small-molecule satisfies Lipinski's Rule of Five.

We further refine through the following steps:

1. Retain structures with at most 1024 residues.
2. Exclude complexes with more than 20 entities.
3. Retaining all the structures containing RNA or DNA entities.
4. Iteratively adding structures containing small-molecules or ions under the condition that all their protein chains belong to new unseen clusters.
5. Iteratively adding multimeric structures under the condition that all the protein chains belong to new unseen clusters. These are further filtered by randomly keeping only 90% of the passing structures.
6. Iteratively adding monomers under the condition that their chain belongs to a new unseen cluster. These are further randomly filtered out by keeping only 60% of the passing structures.

This results in a total of 398 structures from PDB in our validation set.

MD data. Given that all entries from the MD datasets correspond to PDB structures released before the validation cutoff date of 2023-06-01, we constructed test sets by greedily adding structures with the smallest number of members within their cluster group. Here, we use the same clusters defined in the general PDB dataset with a sequence identity threshold of 0.4. Trajectories from the selected clusters and all of their cluster members are then removed from the training set across all 3 MD datasets. This procedure is repeated until we achieve a desired test set of 40 complexes per dataset.

A.2 Binding Affinity Data

We construct a large-scale dataset for training and evaluating our model, comprising both continuous affinity measurements (e.g., K_i, K_d, IC_{50}, AC_{50}, EC_{50}, XC_{50}) and binary labels (binder vs. decoy). The dataset integrates information from a diverse set of public sources:

- **PubChem (1.8.1):** A large public repository maintained by the NIH that contains bioactivity data across a broad range of targets and compounds [Kim et al., 2023].
- **ChEMBL (v34):** A manually curated database of bioactive molecules with drug-like properties, containing standardized binding and functional assay data [Zdrazil et al., 2024].
- **BindingDB:** A public database of measured binding affinities for protein–ligand interactions, with a strong emphasis on K_i and K_d values from medicinal chemistry studies [Liu et al., 2007].

bioRxiv preprint doi: https://doi.org/10.1101/2025.06.14.659707; this version posted June 18, 2025. The copyright holder for this preprint (which was not certified by peer review) is the author/funder, who has granted bioRxiv a license to display the preprint in perpetuity. It is made available under aCC-BY 4.0 International license.

- **CeMM Fragment Dataset:** A dataset of fragment-screening results containing validated binders from fragment-based drug discovery campaigns [Offensperger et al., 2024].
- **MIDAS Metabolite Data (University of Utah):** A set of small-molecule metabolites tested in biochemical binding assays by the University of Utah [Hicks et al., 2023].

Since our model leverages protein–ligand complex structures as input, it is essential to ensure the structural quality of the training data. To this end, we apply a filtering strategy at the assay level that avoids introducing selection bias. For each assay, we compute the average predicted interface pTM (ipTM) score across a set of 10 random binders using the Boltz-2 confidence module. Assays are retained only if this average exceeds 0.75, ensuring that the dataset contains structurally reliable examples suitable for structure-based learning.

We use the ChEMBL Structure Pipeline [Bento et al., 2020] to standardize all ligand molecules across the datasets. This pipeline applies a series of cheminformatics preprocessing steps designed to ensure consistency. The resulting standardized SMILES are then used as input to Boltz-2.

A.2.1 Affinity values

We curate continuous affinity data primarily from ChEMBL and BindingDB, using the following filtering and standardization steps:

ChEMBL data extraction. We extract binding measurements from ChEMBL using the following criteria:

- Filter to confidence score equal 9 (maximum confidence) to retain high-quality structure–activity annotations.
- Target type restricted to `SINGLE PROTEIN` to train on high-quality structures.
- Filter to biochemical or functional assays.
- Filter to affinity measurements with standard type in {'Ki', 'Kd', 'IC50', 'XC50', 'EC50', 'AC50'}.
- Exclude sources flagged as unreliable.
- Parse and retain protein mutation annotations when available.
- Store both the assay ID and activity qualifier for downstream processing.

BindingDB data extraction. We retain only records not already covered by ChEMBL, using the following protocol:

- Use the BindingDB DOI identifier as the assay ID.
- Exclude proteins that report more than 1 chain.
- Retain associated activity qualifiers.
- Parse the protein sequence reported by BindingDB.

General curation. Across both datasets, we:

- Remove PAINS (Pan-Assay Interference Compounds) to eliminate molecules known to produce assay artifacts or interfere with diverse biochemical readouts.
- Filter out molecules with more than 50 heavy atoms.
- Partition the curated affinity data into two subsets: 1) hit-to-lead affinity values, representing optimization-stage datasets, and 2) hit affinity values representing earlier-stage binding screens. This separation allows us to handle affinity values with > qualifiers appropriately: in hit discovery, they are treated as decoys, while in hit-to-lead, they are interpreted as censored measurements, reflecting the uncertainty of whether the compound is a weak binder or a true non-binder.

bioRxiv preprint doi: https://doi.org/10.1101/2025.06.14.659707; this version posted June 18, 2025. The copyright holder for this preprint (which was not certified by peer review) is the author/funder, who has granted bioRxiv a license to display the preprint in perpetuity. It is made available under aCC-BY 4.0 International license.

Hit-to-lead curation. For optimization-stage assays, we apply stricter filters to minimize the noise from these assays:

- Convert all affinity values to logarithmic scale with 1 μM as the reference unit.
- Remove assays with low average pairwise Tanimoto similarity (< 0.25), retaining those aligned with hit-to-lead settings where actives are structurally related.
- Exclude assays with fewer than 10 data points, to further focus the model on fitting affinity differences of similar molecules and not global trends.
- Discard assays with low activity standard deviation (< 0.25), as they do not help to understand activity cliffs.
- Exclude assays with fewer than 10 unique activity values or where the unique-to-total ratio is less than 0.2, as these are likely to come from low-accuracy assays.
- Discard data with qualifiers <.
- Discard data with qualifiers > and activity value $<$ 10 µM.
- Remove assays containing any activity value $< 10^{-6}$ μM, as these often indicate incorrectly reported units or annotation errors.

Hit-discovery curation. For screening assays:

- Retain only assays with at least 100 data points.
- Retain chemically diverse assays (average pairwise Tanimoto similarity < 0.25), discarding hit-to-lead assays with low diversity.
- Label as *inactive* all entries with $>$ qualifiers, as *active* those with $=$ and affinity $<$ 2.0 µM and discard everything else.

Despite our effort, our affinity value curation only scratches the surface of what is possible for constructing high-quality training datasets. Future work could pursue more rigorous standardization by, for instance: (1) applying the Cheng–Prusoff equation to convert inhibition assay values (e.g., IC_{50}) into K_i estimates for more direct comparability; (2) performing deeper assay-level vetting to exclude data from cell-based assays, low-purity protein preparations, or other sources known to introduce noise; and (3) further removing artifacts and confounding signals through advanced filtering or metadata-driven heuristics. However, such refinement is very challenging in practice, as assay metadata are often inconsistently reported, difficult to parse, and require close collaboration with domain experts deeply familiar with the biological and experimental nuances of each assay.

A.2.2 Binary labels

Binary classification data are derived primarily from HTS assays in PubChem and supplemented with fragment, metabolite binding data, and a synthetically generated set of decoys.

PubChem HTS curation. We apply the following filters to construct a reliable binary dataset:

- Retain only assays with at least 100 tested compounds.
- Retain assays with a hit rate (actives/total) < 0.1.
- For each (protein sequence, SMILES) pair, we query PubChem for matching entries that report an affinity value measurement and are explicitly labeled as Active. Only compounds meeting both criteria are retained; all others are discarded. Through cross-referencing with available confirmatory (secondary) screens, we estimate that approximately 40% of the compounds labeled as actives in high-throughput primary screens may be false positives.

bioRxiv preprint doi: https://doi.org/10.1101/2025.06.14.659707; this version posted June 18, 2025. The copyright holder for this preprint (which was not certified by peer review) is the author/funder, who has granted bioRxiv a license to display the preprint in perpetuity. It is made available under aCC-BY 4.0 International license.

- Remove PAINS compounds.
- Subsample the decoy set to achieve an approximate 1:9 ratio of binders to decoys per assay.

CeMM Fragment. We apply the following filters to construct a reliable dataset:

- Remove all fragments labeled with low confidence (score = 1).
- Label as binders all fragments with medium or high confidence (scores = 2 or 3).
- Label as inactives all fragments explicitly marked as decoys (score = 0).
- Subsample the decoy set to achieve an approximate 1:9 ratio of binders to decoys per assay.

Synthetic decoys. To construct a reliable set of synthetic decoys for binary classification, we apply the following procedure:

- Each hit-to-lead compound with an experimentally measured affinity is paired with a single decoy molecule.
- Decoys are sampled from the pool of hit-to-lead molecules to ensure distributional consistency between binders and decoys. This avoids trivial shortcuts where the model could distinguish actives from decoys based solely on distributional differences, rather than true binding signal.
- Assuming that hit-to-lead compounds are selective, we minimize the likelihood of false negatives by sampling decoys from molecules with a Tanimoto similarity < 0.3 to any known binder of targets belonging to the same 90% sequence identity cluster as the current target. This constraint reduces the chance of accidentally including active compounds as decoys.

Binary label curation poses unique challenges, particularly when integrating data from high-throughput screening (HTS). Although HTS datasets are valuable due to their scale and realistic chemical diversity, they are susceptible to multiple sources of systematic noise and artifacts. Examples include false positives arising from promiscuous binding (e.g., PAINS compounds or colloidal aggregators), interference artifacts such as luciferase inhibition or fluorescent signal quenching, and biological noise introduced through cell-based assays where the measured signal may not directly reflect binding to the intended protein target. Our current strategy—matching binary labels to corresponding affinity measurements—provides an initial filter but does not fully ensure reliability or biological relevance. For instance, matched affinity data often stems from secondary assays whose quality or target specificity we have not systematically validated. Achieving a robust binary curation would require deeper metadata interrogation to confirm assay quality, target specificity, and orthogonal validation outcomes. However, inconsistent metadata annotation and reporting across public repositories substantially complicate such efforts, necessitating close collaboration with experimentalists and domain specialists who possess detailed knowledge of assay methodologies and underlying biology.

bioRxiv preprint doi: https://doi.org/10.1101/2025.06.14.659707; this version posted June 18, 2025. The copyright holder for this preprint (which was not certified by peer review) is the author/funder, who has granted bioRxiv a license to display the preprint in perpetuity. It is made available under aCC-BY 4.0 International license.

B Model

B.1 Tokenization and Featurization

Shared across all modules are the tokenization of the biomolecular complexes and the featurization of each atom and each token.

Tokenization. We use the following tokenization scheme: Every protein is tokenized at the amino-acid level, every RNA and DNA at the nucleotide level, and other biomolecules are tokenized at the atomic level. Unlike AlphaFold3, Chai-1, and Boltz-1, where non-canonical amino acids and nucleotides are tokenized at the atomic level, we keep them as a single token as well.

Featurization. Compared to Boltz-1, Boltz-2 has the following additional features that are given as input to the model. At the single token level, a cyclic flag distinguishing cyclic polymers from acyclic ones, a modified flag distinguishing non-canonical amino acids or nucleotides, a one-hot encoding for different experimental method types, and a molecular type feature encoding whether a token belongs to a protein, DNA, RNA, or other. At the pairwise token level, a bond type feature distinguishes the order/aromaticity of bonds between pairs of tokens. Moreover, the relative positional encoder was modified to have cyclic-offset positional encodings for cyclic polymers (similar to Rettie et al. [2025]) and only have the relative chain encoding between symmetric chains (similar to ProteinX [Chen et al., 2025]). Finally, for sequences in the MSA, we add an additional binary feature to every token representing whether or not it is part of a paired sequence.

B.2 Trunk and Denoising Modules Architecture

Trunk Module. At high-level, the architecture of the model in the trunk is similar to that of Boltz-1 with a few exceptions:

1. Boltz-2 utilizes a template module similar to AlphaFold3.
2. The number of PairFormer layers are increased from 48 to 64.
3. For a majority of the trunk, we employed mixed-precision training (using `bfloat16`) and `trifast` kernels for triangular attention operations. This allowed us to scale the crop size at training time from 512 (in Boltz-1) to 768 (similar to AlphaFold3).

Denoising Module. We used the same denoising module as Boltz-1. The denoising module was trained in `float32` precision due to instabilities observed at lower precision.

Physical quality. Deep learning based co-folding models such as AlphaFold3, Chai-1 and Boltz-1 suffer from significant physical issues with the poses they generate. These include the presence of chain clashing hallucinations as well as other issues including steric clashes between atoms, slightly incorrect bond lengths and angles, incorrect stereochemistry at chiral centers and stereobonds and aromatic rings predicted to be non-planar. We recently introduced Boltz-steering, a new inference-time technique that, when applied with a set of physics-based potentials on top of Boltz-1 gave rise to Boltz-1x keeping the original geometric accuracy while solving many of these physical issues. Boltz-2 adopts the steering potentials we proposed in Boltz-1x with tuned hyperparameters and a normalization of each potential by the number of elements on which they are applied. Additionally, as presented in the next section, we integrated additional potentials to improve controllability.

bioRxiv preprint doi: https://doi.org/10.1101/2025.06.14.659707; this version posted June 18, 2025. The copyright holder for this preprint (which was not certified by peer review) is the author/funder, who has granted bioRxiv a license to display the preprint in perpetuity. It is made available under aCC-BY 4.0 International license.

B.3 Controllability

B.3.1 Method conditioning

Boltz-2 is trained with structural data generated with a variety of different experimental methods, including but not limited to: X-ray diffraction, electron microscopy, solution NMR, solid-state NMR, molecular dynamics, distillation from AlphaFold2 and distillation from Boltz-1. As structure prediction methods are reaching experimental accuracy, it is important to teach them to understand the different properties that structures coming from different experimental methods have.

Therefore, at training time, we condition the model to the experimental method to obtain the specific structure by giving its experimental method as input in the single token representations. At inference time, users can decide on which experimental method to use to condition the model's prediction. As shown in Section 5.2, this conditioning does indeed have an effect on the resulting structure distribution, leading it closer to the one obtained with the desired experimental technique.

B.3.2 Templates conditioning and steering

Templates allow the users to feed the model the structures of related biomolecular complexes that might help with the prediction of the complex under analysis. While not very effective when fed with structures within the model's training set, templates can be particularly useful in settings where users have access to unseen relevant structures or have a strong prior on the complex structure having a particularly similar fold.

While Boltz-1 does not support template conditioning, AlphaFold3 and Chai-1 integrate it. However, they only allow for single chains to be used as templates, and they do not enforce the model to necessarily respect the given template, often leading to no improvements from the template addition. In Boltz-2, we improve on these two fronts: we allow for multimeric templates and we allow the user to strictly enforce that templates are respected via a Boltz-steering potential. Similar to previous models, at this point, we only allow for potentials within protein chains.

Template conditioning. We first produce template hits for all monomeric protein chains. During training, we then group the templates by their PDB ID so that if chains A and B yield two templates of the same PDB ID, these will be grouped together and used as a multimeric template. Note that we only do this over protein chains. Following AlphaFold3, we always sample 0 to 4 templates per chain. We do so by keeping a counter for each chain and using template groups as defined above to assign templates to chains with non-zero counters. Whereas previous approaches limit the template mask to only be non-zero along the diagonal (i.e, monomeric templating), in our approach, templates with the same PDB ID are visible to one another during template encoding.

Template steering. If the user desires to enforce the potential beyond what the conditioning does, we devised an inference time Boltz-steering potential that pushes the reverse diffusion to place the portion of the structure corresponding to the template (which can be a subset of a chain, such as a pocket, a domain or a loop) to have a structure within α_{cutoff} Å of the given template.

For a template with reference atoms $S_{\text{template atoms}}$, we define the potential as follows.

$$E_{planar}(\mathbf{x}) = \sum_{i \in S_{\text{template atoms}}} \max\left(\left\|\mathbf{x}_i - \mathbf{x}_i^{\text{ref}}\right\| - \alpha_{\text{cutoff}}, 0\right)$$

where $\mathbf{x}_i^{\text{ref}}$ is the position of reference atom i after aligning the template to the predicted coordinates.

B.3.3 Contacts and pocket conditioning and steering

From experimental data or intuition, structural biologists often have hypotheses about which residues within a complex might interact or which site of a polymer another molecule might bind to. We will define a contact as the specification of a distance constraint between two tokens (residues/atoms),

bioRxiv preprint doi: https://doi.org/10.1101/2025.06.14.659707; this version posted June 18, 2025. The copyright holder for this preprint (which was not certified by peer review) is the author/funder, who has granted bioRxiv a license to display the preprint in perpetuity. It is made available under aCC-BY 4.0 International license.

and a pocket as the specification of a distance constraint between one or more tokens and a separate chain/molecule.

Although it did report results on a separately trained model with pocket conditioning, AlphaFold3's publicly available model does not support any distance constraint specification. Boltz-1 supports the specifications of pocket conditioning, Chai-1 supports both pocket and contact conditioning with flexible distance cutoff. In Boltz-2, we also support both pocket and contact conditioning with flexible distance cutoff. These are added not only like previous models with feature conditioning, in which case the model sometimes takes samples that do not respect these conditions, but also with steering potentials to enforce them.

Contact and pocket conditioning. Boltz-1 defined pocket conditioning via features fed into the single token representation. This, however, has the limitation that at most one restraint can be specified. Therefore, in Boltz-2, we feed contact and pocket conditioning as pairwise features between tokens. These features consist of a one-hot encoding of the contact type and an encoding of the distance. The contact type is selected among: no restraint was specified, some were specified but this was not selected, this pair has a pocket-to-binder relationship, this pair has a binder-to-pocket relationship, this pair has a contact relationship (takes precedence). The encoding of the distance d, constrained to be $4\text{Å} \leq d \leq 20\text{Å}$, is encoded as a concatenation of the normalized distance $(d-4)/16$ and its Fourier embedding with a fixed set of randomly sampled bases.

Contact and pocket steering. When defining a contact or a pocket, these can be interpreted as a relationship between two sets of atoms, S_A and S_B, where our goal is to ensure that the smallest distance between the atoms in these sets is less than a threshold r_{AB}. To do this, we define a time-dependent potential function as follows.

$$E^t_{\text{Contact(A,B)}}(\mathbf{x}) = \frac{\sum_{(i,j)\in S_\text{A}\times S_\text{B}} \exp\left(-\lambda^t_{\text{union}} \max\left(\|\mathbf{x}_i - \mathbf{x}_j\| - r_{AB}, 0\right)\right) \max\left(\|\mathbf{x}_i - \mathbf{x}_j\| - r_{AB}, 0\right)}{\sum_{(i,j)\in S_\text{A}\times S_\text{B}} \exp\left(-\lambda^t_{\text{union}} \max\left(\|\mathbf{x}_i - \mathbf{x}_j\| - r_{AB}, 0\right)\right)}$$

where λ^t_{union} is a time-dependent parameter which monotonically increases as t goes from 1 to 0. As $t \to 0$, the potential biases the model towards conformers where all pairs of atoms are within a distance of r_{AB}, and as $t \to \infty$, the potential biases the model towards conformers where any pair of atoms is within a distance of r_{AB} to enable more flexibility in the ligand conformation within the pocket.

B.4 Confidence Module Architecture

The confidence module of Boltz-2 has an architecture that resembles that of AlphaFold3's confidence model. Instead, Boltz-1 has a significantly more expensive confidence model that included a trunk of the same size of the structure prediction trunk (48 layers of PairFormer plus AtomEncoder and MSA modules), which is initialized with the weights of the structure prediction trunk and includes inputs from the DiffusionTransformer final representations. While this larger architecture provides some improvement over the simpler architecture of AlphaFold3 and Boltz-2 confidence model, it comes at a significant cost.

Therefore, we opted for a faster architecture, using eight PairFormer layers (versus the four of AlphaFold3) on top of the final pair token representation of the structure trunk and the encoding of the predicted coordinates. Unlike previous models, we found it beneficial to divide the final heads predicting the PDE and PAE logits into two separate layers, one making the prediction for pairs of tokens within the same chain/molecule and one making the prediction for pairs across different chains/molecules.

B.5 Affinity Module Architecture

One of the core challenges in drug discovery is accurately determining whether a small molecule binds to a given protein target and quantifying the strength of this interaction. Boltz-2 enhances this capability through a specialized affinity module designed to address two key prediction tasks:

bioRxiv preprint doi: https://doi.org/10.1101/2025.06.14.659707; this version posted June 18, 2025. The copyright holder for this preprint (which was not certified by peer review) is the author/funder, who has granted bioRxiv a license to display the preprint in perpetuity. It is made available under aCC-BY 4.0 International license.

Algorithm 1: AFFINITY MODULE

Input: trunk representation `z_trunk` after 5 recycling
Input: trunk input features `s_inputs`
Input: distogram of the predicted token coordinates $D_{i,j}$

```
// Initialize single and pair representation
z ← LinearNoBias(LayerNorm(z_trunk))
z += LinearNoBias(s_inputs[:, :, None]) + LinearNoBias(s_inputs[:, None, :])
z += PairwiseConditioner(z, LinearNoBias(one_hot(D_{i,j})))
z += PairFormerModule(z, pair_mask=protein_ligand_mask + intra_ligand_mask)
g = MeanPooling(z, mask = protein_ligand_mask + intra_ligand_mask * (1 - Id))
g = ReLU(Linear(ReLU(Linear(g)))
// Predict affinity
binding_likelihood = SoftMax(Linear(ReLU(Linear(ReLU(Linear((g)))))))
affinity_value = Linear(ReLU(Linear(ReLU(Linear((g)))))))
```

Output: `binding_likelihood, affinity_value`

1. **Binding likelihood**: Predicting the likelihood that a small molecule will bind to a specific protein target.
2. **Affinity value**: Quantifying the strength of the interaction between a small molecule and a protein target, measured similar to the half-maximal inhibitory concentration (IC_{50}).

The binding likelihood head is optimized for identifying potential hits across diverse molecular screening scenarios. In contrast, the affinity value head is specifically tailored to guide hit-to-lead optimization by discerning subtle variations in binding strengths among structurally related molecules targeting the same protein. Algorithm 1 details the full implementation of this affinity module.

B.5.1 Affinity Module

The affinity module operates on the structural predictions from Boltz-2, specifically utilizing the input single representations s_{inputs} and the final pair representation $\mathbf{z}_{ij}$ obtained after five recycling iterations. Coordinates fed into the module are selected as the top-ranked structure from five samples generated over 200 diffusion steps each, ranked according to their protein-ligand ipTM-score.

At its core, the affinity architecture comprises a Pairformer model designed to process the interaction pair representations, masking out intra-protein interactions to focus exclusively on protein-ligand interface details. To achieve a scalar representation for the downstream affinity prediction, the module performs mean pooling over all pairwise interactions.

Following pooling, two dedicated multi-layer perceptron (MLP) heads produce distinct outputs: one providing logits for binding likelihood estimation and another regressing continuous affinity values.

B.5.2 Affinity Model Ensemble

To improve robustness and overall performance, we train two affinity models with distinct hyperparameters. The models differ in binder-to-decoy loss weighting ($\lambda_{\text{focal}} = 0.8$ vs. 0.6), the number of transformer layers (4 vs. 8), and training duration (one is trained longer while the other is early-stopped). This diversity not only enhances predictive accuracy through ensembling but also serves an important role in downstream molecule generation. When using SynFlowNets for optimization, there is a risk of over-optimizing against a single model's reward signal. To mitigate this, we use the second model as an independent reference for final filtering, reducing the likelihood of reward hacking and introducing a more stable selection criterion.

We ensemble the models as follows:

- For binary classification, we take the average of the predicted binding likelihoods.

bioRxiv preprint doi: https://doi.org/10.1101/2025.06.14.659707; this version posted June 18, 2025. The copyright holder for this preprint (which was not certified by peer review) is the author/funder, who has granted bioRxiv a license to display the preprint in perpetuity. It is made available under aCC-BY 4.0 International license.

- For affinity regression, we apply a calibrated ensembling strategy. We first compute the mean predicted affinity between models and then apply a molecular weight correction of the form

$$\hat{y} = C_0 \cdot (y_1 + y_2) + C_1 \cdot \mathrm{MW}_{\mathrm{binder}} + C_2,$$

where y_1 and y_2 are the predictions of the two models, C_0, C_1, and C_2 are fitted in the holdout validation set and $\mathrm{MW}_{\mathrm{binder}}$ is the molecular weight of the binding small molecule.

In Table 3, we report the differences in hyperparameters between the two affinity models used in the ensemble. Both models are trained across 128 A100 GPUs using the AdamW optimizer with a weight decay of 0.001 and a learning rate of 0.0001.

Table 3: Extra hyperparameters that differ between the two affinity models trained for the ensemble.

	**Ensemble member 1**	**Ensemble member 2**
PairFormers layers	8	4
λ_{focal}	0.8	0.6
Training samples ($\cdot 10^6$)	55	12.5

B.6 SynFlowNet Ligand-Generation Model

For SynFlowNet, we use a similar setup to that of Cretu et al. [2024]. The Markov Decision Process (MDP) traversed by the agent represents partial molecules, with the action space comprising both uni- and bi-molecular reactions and of a set of building blocks. The agent sequentially constructs each trajectory by combining these elements. The forward policy P_F is parameterized by a Graph Transformer model [Yun et al., 2019], while the backward policy P_B is a uniform distribution over the backward actions. We employed the trajectory balance loss [Malkin et al., 2022]. All model and training hyperparameters are detailed in Table 4.

Table 4: Model and training hyperparameters for our SynFlowNet molecular generation model.

**Hyperparameter**	**Value**
Numbr of training steps	11,000
Training batch size	64
Replay buffer warmup	500
Maximum trajectory length	3
Reward function exponent (β)	36
Random action probability (exploration)	0.20
Target policy soft update parameter (τ)	0.99
Training loss	Trajectory Balance
Backward policy P_B type	Uniform
Optimizer	Adam
Forward policy P_F learning rate	10^{-4}
P_F learning rate decay	$2,000$
Normalizing constant Z learning rate	10^{-3}
Z learning rate decay	50,000
Graph transformer embedding size	128
Graph transformer depth	4
Graph transformer number of heads	2
Action space reactions (number & source)	105 (Hartenfeller)
Action space building blocks (number & source)	240,278 (Enamine REAL)
Action space building blocks embedding	Morgan fingerprint (1024)

bioRxiv preprint doi: https://doi.org/10.1101/2025.06.14.659707; this version posted June 18, 2025. The copyright holder for this preprint (which was not certified by peer review) is the author/funder, who has granted bioRxiv a license to display the preprint in perpetuity. It is made available under aCC-BY 4.0 International license.

C Training

C.1 Structure and confidence training

C.1.1 Main differences with Boltz-1

At a high level, the structure and confidence training phases are similar to those from Boltz-1, but there are a few key differences.

MSA sampling In order to promote robustness of the model with regards to low-quality MSAs, at training time, we do not select MSA sequences greedily, but we rather sample them randomly among the top 16k hits. Moreover, given the recent progress on protein design models that rely on single-sequence folding predictions (without language model embeddings) [Pacesa et al., 2024, Cho et al., 2025], we aim at improving the model performance in this setting by randomly dropping all of the MSA of a complex in 5% of training iterations.

Ensemble supervision While Boltz-1 supervises on only a single structure, Boltz-2 integrates multiple samples from experimental ensembles and MD trajectories. Structure supervision happens at two stages in the model: the trunk's histogram output and the denoising module. Given an ensemble with K structures, we aggregate the one-hot encoded distograms of all K conformers and perform a weighted multi-class cross-entropy. For the coordinate noising and denoising supervision, we randomly sample at each training iteration one conformer to be used.

B-factor On top of supervising the final pairwise representation of the trunk to predict the relative distances between pairs of tokens, we additionally supervise each token's single representation to predict the B-factor of its representative atom. MD structures are supervised by computing the B-factor from the RMSF values computed over the trajectory, as given by [Kuzmanic et al., 2014]:

$$B = \frac{8\pi^2}{3} \cdot RMSF^2$$

C.1.2 Controllability Training

Below is a description of the way that we sample templates, contact and pocket information at training time. Note that this sampling occurs after the cropping and therefore only applies to the entities and tokens that are present in the cropped structure.

Template sampling Templates during training are sampled independently for each individual entity. 60% of the times no templates are selected for the selected chain. In the remaining 40% of the times, a random number of templates between 1 and min(1, # templates) is chosen. These templates are selected at random between the top 20 template hits for that particular entity. Multimeric templates are constructed by adding additional chains that are in the same PDB id and are template hits for chains in the complex that are still missing templates. No multimeric templating is done for symmetric entities to avoid issues with the chain mapping.

Contacts sampling For each training example, we sample the number of contacts to add from a geometric distribution starting from 0 with $p = 0.85$. For each contact, we first sample the contact cutoff (maximum distance) between d=4Å and 20Å with a probability proportional to 1/d. Then, if there are multiple chains, we select a pair of tokens between different chains that have at least a pair of atoms within the cutoff. The sampling is done by selecting first a chain at random, enumerating all possible contacts and then selecting at random between the contacts. If there is only one chain, then we look for contacts between tokens that are at least 8 residues apart.

bioRxiv preprint doi: https://doi.org/10.1101/2025.06.14.659707; this version posted June 18, 2025. The copyright holder for this preprint (which was not certified by peer review) is the author/funder, who has granted bioRxiv a license to display the preprint in perpetuity. It is made available under aCC-BY 4.0 International license.

Pocket sampling Similarly, to contact sampling, for each training example, we sample the number of pockets to specify from a geometric distribution starting from 0 with $p = 0.85$. For each pocket, we select a ligand at random if present, otherwise an arbitrary chain. Then, we sample the pocket cutoff (maximum distance) between d=4Å and 20Å with a probability proportional to 1/d. Then, the number of pocket tokens to specify for the pocket in question is sampled with a geometric distribution starting from 1 with $p = 0.7$. These pocket tokens are then sampled at random from the available ones.

C.1.3 Training stages

Table 5 shows the parameters used in the different stages of structure prediction training of the model. Most of the training happens at crop size of 384 tokens (atom crop size is always computed to be $\tilde{9}$x the number of tokens) with more limited stages expanding this gradually to 512, 640, and 768. At the final stage, we exclude both the MD and the Boltz-1 distillation data to maintain only the highest quality datapoints. The molecular dynamics data was not included in the first stage of training due to project timing, we would expect bigger gains in the model's ability to model dynamics had this data been integrated in the model earlier.

Training stage	Learning rate	Crop size	Training steps	Include MD	Include Boltz-1 distillation
1st	1e-3	384	88k	No	Yes
2nd	5e-4	512	4k	Yes	Yes
3rd	5e-4	640	4k	Yes	Yes
4th	5e-4	768	1k	No	No

Table 5: Overview of the different stages for the structure prediction training.

Confidence training was performed as a single stage using a crop size of 512 tokens and only trained on PDB data. In order to make the confidence model more robust to different inference hyperparameters, at every training iteration, we randomly sampled the number of inference steps between [20, 50, 200] and the diffusion step scale between [1.0, 1.1, 1.2, 1.3, 1.4, 1.5].

C.1.4 Architecture, Training and Inference Hyperparameters

Tables 6 and 7 record some of the hyperparameters of Boltz-2's architecture, training and inference procedures that differ from Boltz-1's and were not previously mentioned in the manuscript. For a full list of the hyperparameters and their precise impact on the model, we recommend the reader to refer directly to the code repository.

Parameter	Value
Max number of MSA sequences during training	8192
Template pairwise dim	64
Num template blocks	2
Training diffusion multiplicity	32
bfactor loss weight	1×10^{-3}

Table 6: Extra model architecture and training hyperparameters that differ from Boltz-1 and were not previously mentioned in the manuscript.

Parameter	Value
sigma_min	0.0001
rho	7
gamma_0	0.8
gamma_min	1.0
noise_scale	1.003
step_scale	1.5

Table 7: Diffusion process hyperparameters that differ from Boltz-1, with the exception of sigma_min we opted for AlphaFold3's default hyperparameters, see Abramson et al. [2024] for more details.

bioRxiv preprint doi: https://doi.org/10.1101/2025.06.14.659707; this version posted June 18, 2025. The copyright holder for this preprint (which was not certified by peer review) is the author/funder, who has granted bioRxiv a license to display the preprint in perpetuity. It is made available under aCC-BY 4.0 International license.

Algorithm 2: POCKET PRE-PROCESSING

```
Input: Protein sequence prot_seq
Input: Ligand smiles { smiles^(1), ..., smiles^(10)}
// Predict structure for the 10 complexes
for i ∈ {1, ..., 10} do
    x^(i) ← Boltz-2-structure(prot_seq, smiles^(i))
end
dist^(i) ← dist(x^(i)[None, protein], x^(i)[ligand, None])
min_dist_protein_to_ligand^(i) ← min(dist^(i), axis=0)
closest^(i) ← argsort(min_dist_protein_to_ligand^(i))[:500]
// Compute overlap matrix
for i, j ∈ {1, ..., 10} do
    overlap[i,j] ← |closest^(i) ∩ closest^(j)| / K
end
// Select structure with highest average overlap
best_idx ← argmax (1/10) Σ_{j=1}^{10} overlap[i,j]
Output: min_dist_protein_to_ligand^(best_idx)
```

C.2 Affinity training

Affinity training occurs after structural prediction, with gradients detached from the structural model to preserve its learned representations. Specifically, the training pipeline consists of the following key components:

1. Efficient pre-processing of protein binding pockets.
2. Cropping of spatial regions around the binding site.
3. Pre-processing of trunk features.
4. Sampling strategy that balances binders and decoys and prioritizes informative assays.
5. Robust loss functions tailored to mitigate the effects of experimental noise.

Each of these components is detailed in the following sections.

C.2.1 Pocket pre-processing

Target-based drug discovery generally assumes that most protein-ligand interactions occur within the binding pocket. To reduce the complexity of training and inference and reduce overfitting, we implement a pocket identification and cropping procedure.

The pocket is identified by computing the minimum atom-wise distance between the ligand and the surrounding protein structure. Specifically, for each target protein, we randomly sample 10 binders from the affinity training dataset and use Boltz-2 with 10 recycling steps, 200 diffusion iterations, and 5 structural samples per complex to predict their structure. We select the most confident structure according to the inter-chain predicted TM-score (ipTM).

From the resulting structures, we derive per-atom distance profiles between the protein and ligand. A consensus-based voting strategy is applied across the 10 binders' structures to select the most likely binding site. The minimum distances from protein atoms to the ligand are cached and used by the *affinity cropper*. More details are provided in Algorithm 2.

C.2.2 Affinity cropper

We propose a cropping algorithm that leverages pre-computed pocket annotations to efficiently crop the complex around the binding site. This approach enables consistent cropping across complexes,

bioRxiv preprint doi: https://doi.org/10.1101/2025.06.14.659707; this version posted June 18, 2025. The copyright holder for this preprint (which was not certified by peer review) is the author/funder, who has granted bioRxiv a license to display the preprint in perpetuity. It is made available under aCC-BY 4.0 International license.

Algorithm 3: Affinity Cropper

```
Input: Token list tokens, minimum distance to the ligand min_dist_protein_to_ligand
Input: max_tokens = 256, max_protein_tokens = 200, neighborhood_size = 10
// Start with all ligand tokens
cropped_tokens ← tokens[mol_type = ligand]
// Min pooling over the residues' atoms
min_dist_res_to_ligand ← MinPooling(min_dist_protein_to_ligand[protein])
res_idx_sorted ← argsort(min_dist_res_to_ligand)
// Add protein tokens around pocket residues
for res_idx in res_idx_sorted do
    Let res_tokens be the entries with res_idx
    Let chain_id be the asym_id of the current residue
    Let chain_tokens be protein tokens with asym_id = chain_id
    // Initialize residue window
    min_idx = max_idx = res_idx
    while len(res_tokens) < neighborhood_size do
        min_idx = min_idx - 1
        max_idx = max_idx + 1
        res_tokens ← all tokens in chain_tokens with res_idx ∈ [min_idx, max_idx]
    end
    Let new_tokens be the entries in res_tokens not in cropped_tokens
    // Check token limits
    if cropped_tokens ∪ new_tokens > max_tokens or (cropped_tokens ∪ new_tokens) ∩
     protein_tokens > max_protein_tokens then
        break
    end
    cropped_tokens ← cropped_tokens ∪ new_tokens
end
Output: cropped_tokens
```

even when all the complex structures are unavailable, and reduces the pre-processing complexity from $\mathcal{O}(\#\text{ complexes})$ to $\mathcal{O}(\#\text{ proteins})$.

The cropping procedure proceeds as follows. First, all ligand tokens are retained. Next, we apply a pocket-centered selection strategy inspired by the Boltz-2 structure model: For each protein token, we use the pocket annotation by selecting the nearest neighbors and apply the usual cropping algorithm with a neighborhood size of 10. We retain 256 tokens with a maximum of 200 protein tokens, to ensure consistency between molecules of different sizes at training.

Full details of the affinity cropper are provided in Algorithm 3.

C.2.3 Feature pre-processing

To reduce computational overhead during training, we pre-compute key structural and representational features. For each protein–ligand complex, we run Boltz-2 structure model with 5 recycling iterations, 200 diffusion steps, and generate 5 candidate structures. The most confident structure based on the interface predicted TM-score (ipTM) is retained for downstream use.

We extract and store the predicted atomic coordinates as well as the trunk pair representation and the cropped token indices. Since the affinity module utilizes only the protein–ligand and intra-ligand pairwise features, we discard the remaining pairwise interactions, and reduce the memory footprint by > 5x.

C.2.4 Training sampler

We design a custom affinity training sampler to enhance the model's ability to learn from the noisy datasets. The sampler is constructed to balance binders and decoys, and to emphasize high-contrast

bioRxiv preprint doi: https://doi.org/10.1101/2025.06.14.659707; this version posted June 18, 2025. The copyright holder for this preprint (which was not certified by peer review) is the author/funder, who has granted bioRxiv a license to display the preprint in perpetuity. It is made available under aCC-BY 4.0 International license.

Algorithm 4: Activity Cliff Sampler

```
Input: Affinity dataset 𝒟 with assay IDs and affinity values
Input: Batch size B = 5
// Preprocessing (done once)
foreach assay a in dataset do
    Let 𝒟_a = {(x_i, y_i) ∈ 𝒟 | assay(x_i) = a}
    Compute Q_1^a = Quantile_0.25({y_i}_{i∈𝒟_a})
    Compute Q_3^a = Quantile_0.75({y_i}_{i∈𝒟_a})
    Set IQR^a = |Q_3^a − Q_1^a|
end
Normalize weights: w_a = IQR^a / Σ_b IQR^b
// Sampling (per batch)
Sample an assay a from 𝒟 using weights w_a
Sample B protein–ligand complexes {x_1, ..., x_B} uniformly from 𝒟_a
return Batch {x_1, ..., x_B}
```

assays that provide valuable learning signals.

During training, we sample from the different data sources with probabilities specified in Table 8. For each sampled source, we construct batches of size 5, enforcing that all samples within a batch come from the same assay. We apply distinct sampling strategies depending on the type of label: datasets with continuous affinity values are treated differently from those with binary binding labels, allowing the model to better adapt to the nature of each supervision signal.

Source	Supervision	Sampling weight
ChEMBL and BindingDB	values	0.25
PubChem small assays	values	0.005
PubChem HTS	binary	0.44
PubChem small assays	binary	0.02
CeMM Fragments	binary	0.03
MIDAS Metabolites	binary	0.005
ChEMBL and BindingDB synthetic decoys	binary	0.25

Table 8: Breakdown of affinity training data sources and their relative sampling weights.

Affinity value sampler. A key challenge in learning from affinity data lies in capturing activity cliffs—subtle, large shifts in binding affinity triggered by small structural modifications to a molecule. To encourage the model to focus on these high-frequency patterns, we sample five complexes coming from the same assay within each batch. This helps the model learn high-frequency variations and allows more complex loss functions as described in Appendix C.2.5.

To prioritize the most informative assays, we introduce an assay-level activity cliff score, defined as the interquartile range (IQR) of affinity values: the difference between the 75th and 25th percentiles of the affinity values. Sampling probabilities over assays are proportional to the activity cliff scores. Full details in Algorithm 4.

Binary label sampler. To improve discrimination between binders and decoys, we construct training batches with a consistent protein context. For each batch (1) we sample uniformly at random a binder from the dataset, (2) identify the associated assay and (3) randomly sample four decoys from the same assay.

C.2.5 Affinity supervision

We jointly train the binary classification and continuous affinity regression heads using the loss functions detailed below.

bioRxiv preprint doi: https://doi.org/10.1101/2025.06.14.659707; this version posted June 18, 2025. The copyright holder for this preprint (which was not certified by peer review) is the author/funder, who has granted bioRxiv a license to display the preprint in perpetuity. It is made available under aCC-BY 4.0 International license.

Affinity value supervision. Affinity measurements are notoriously noisy, with variability arising both from experimental replicates and inter-laboratory differences. Furthermore, IC_{50} values are highly sensitive to assay conditions, including substrate concentration and assay type, and may not be directly comparable across datasets [Landrum and Riniker, 2024]. While the Cheng–Prusoff equation is commonly used to convert IC_{50} to K_i by correcting for substrate concentration, this correction is frequently infeasible due to missing metadata (eg. the substrate concentration or K_m).

To address this, we introduce a supervision strategy based on pairwise differences of affinity values within the same assay. This difference-based formulation implicitly cancels out assay-specific confounding factors, such as those corrected by the Cheng–Prusoff equation.

We use a Huber loss—a quadratic loss for small errors $< \delta$, but linear otherwise—to supervise both the affinity differences and the absolute values, with a weighted combination of the two. Many experimental affinity values are reported with inequality qualifiers (e.g., '=' or '>') rather than exact values. For entries with qualifier '>', we interpret the label as a lower bound and include the example in the loss only if the model prediction is *lower* than the reported value, both for the absolute affinity term and for its pairwise differences. This censor-aware supervision ensures that the model is not penalized for correct directional predictions when ground truth values are bounds rather than measurements.

The resulting loss functions are:

$$\mathcal{L}_{abs}(y, \hat{y}, \mathrm{s}) = \begin{cases} \mathrm{Huber}(y, \hat{y}; \delta = 0.5) & \text{if s is } = \\ \mathrm{Huber}(y, \hat{y}; \delta = 0.5) \cdot \mathbb{1}[\hat{y} < y] & \text{if s is } > \end{cases}$$

$$\mathcal{L}_{dif}(y_1, y_2, \hat{y}_1, \hat{y}_2, \mathrm{s}_1, \mathrm{s}_2) = \begin{cases} \mathrm{Huber}(y_1 - y_2, \hat{y}_1 - \hat{y}_2; \delta = 0.5) & \text{if } \mathrm{s}_1 \text{ is } =, \mathrm{s}_2 \text{ is } = \\ \mathrm{Huber}(y_1 - y_2, \hat{y}_1 - \hat{y}_2; \delta = 0.5) \cdot \mathbb{1}[\hat{y}_1 - \hat{y}_2 > y_1 - y_2] & \text{if } \mathrm{s}_1 \text{ is } =, \mathrm{s}_2 \text{ is } > \\ \mathrm{Huber}(y_1 - y_2, \hat{y}_1 - \hat{y}_2; \delta = 0.5) \cdot \mathbb{1}[\hat{y}_1 - \hat{y}_2 < y_1 - y_2] & \text{if } \mathrm{s}_1 \text{ is } >, \mathrm{s}_2 \text{ is } = \\ 0 & \text{if } \mathrm{s}_1 \text{ is } >, \mathrm{s}_2 \text{ is } > \end{cases}$$

where s, s_1 and s_2 are the affinity qualifier of the ground truth measurement, and $\mathbb{1}$ is the indicator function, which returns 1 if the condition holds, otherwise 0.

Binary label supervision. For binary binding classification, we use a focal loss with $\gamma = 1$, along with a balancing coefficient λ_{focal} to weight the contribution of positive and negative samples:

$$\mathcal{L}_{\mathrm{binary}} = \mathrm{Focal}(\mathrm{logits}, \gamma = 1, \alpha = \lambda_{focal})$$

Overall loss. The final training objective is a weighted sum of the three components:

$$\mathcal{L}_{\mathrm{total}} = 0.9 \cdot \mathcal{L}_{\mathrm{dif}} + 0.1 \cdot \mathcal{L}_{\mathrm{abs}} + \mathcal{L}_{\mathrm{binary}}$$

C.3 Generative ligand optimization with SynFlowNet

Binding affinity is among the most critical properties to optimize in the early stages of target-based drug discovery. Over the years, multiple generative models have been developed to generate novel ligands for early hit identification [Segler et al., 2018, Jensen, 2019, Du et al., 2024], but these methods often suffer from synthesizability issues and from the lack of a robust and fast scoring function to optimize against. Hence, generative models would often adversarially exploit the scoring function and generate non-sensical molecules, for example, by simply concatenating high-reward functional groups together [Renz et al., 2020, Langevin et al., 2022, Walters, 2024a,b].

Here we combine the proposed Boltz-2 model with SynFlowNet [Cretu et al., 2024], our recently published synthesis-aware molecular generator, to address these longstanding challenges. First, Boltz-2 offers the appropriate speed/accuracy trade-off needed for robust scoring functions. Our results in Section 5.3 show that Boltz-2 approaches FEP accuracy on public benchmarks. With an inference time

bioRxiv preprint doi: https://doi.org/10.1101/2025.06.14.659707; this version posted June 18, 2025. The copyright holder for this preprint (which was not certified by peer review) is the author/funder, who has granted bioRxiv a license to display the preprint in perpetuity. It is made available under aCC-BY 4.0 International license.

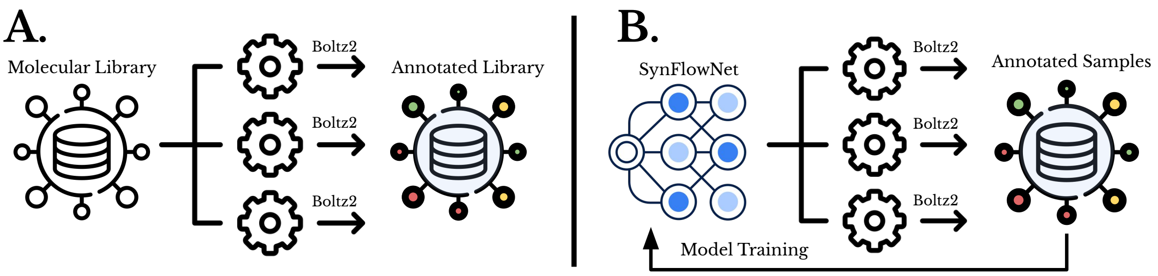

Figure 9: Depiction of two virtual screening strategies. A) Fixed-Library Virtual Screen: We start from a fixed molecular library and leverage high-performance computing infrastructure to accelerate the screening time by employing multiple paralellized Boltz-2 workers. B) Generative Virtual Screens: We make use of a molecular generation model to sample batches of compounds to be annotated by Boltz-2. The compounds gradually build a sampled annotated library which is dynamically used to asynchronously train the generative model and generate additional compounds.

of approximately 20 seconds per ligand, integrating Boltz-2 with modern parallel high-performance computing infrastructure enables the screening of hundreds of thousands of compounds per day. SynFLowNet, on the other end, offers the possibility of de novo molecular generation directly via synthetizable routes, naturally handling purchasability for downstream experimental validations. We combine these two models to perform a *Generative Virtual Screening* of ultra-large scale libraries of purchasable compounds (Enamine 76B REAL space) against a specific protein target. As opposed to Fixed-Library Virtual Screening, where a pre-assembled set of molecules is scored to identify potential hits, generative screens enable further exploration into the molecular space, far outside off-the-shelf libraries, by simultaneously training a parameterized sampler and building an annotated set of candidates (Figure 9).

The main score used for compound selection combines both the binding likelihood and the affinity value predictions of the first ensemble model (see Appendix B.5.2):

$$\text{score} := \max\left(\frac{-\text{affinity}+2}{4}, 0\right) * \text{likelihood}$$

where the affinity value prediction is approximately normalised and lower-bounded to 0.

The SynFlowNet model uses this score as a reward function. The computational delay to obtain a numerical score for each molecule using Boltz-2 is approximately 20 seconds. With a batch size of 64, utilizing a single scoring instance would introduce significant latency in the training of the generative model. To mitigate this limitation, we implemented an asynchronous training paradigm: the SynFlowNet model continuously samples new trajectories from its current policy P_F and submits them to a Last-In, First-Out (LIFO) Reward Queue. Concurrently, it samples batches of training data from an annotated Replay Buffer, which comprises all previously scored trajectories. An array of Boltz-2 scorers operates in parallel, retrieving new trajectories from the Reward Queue and depositing the annotated results into the Replay Buffer. A training batch pulled from the Replay Buffer consists of 50% trajectories uniformly sampled across the buffer (off-policy) and 50% of the newest trajectories added to the buffer (on-policy). For the screen presented in Section 5.4, we employed an array of 60 parallel Boltz-2 workers, each executing on a single H100 GPU. It is anticipated that the SynFlowNet policy will re-sample previously encountered molecules multiple times. To obviate unnecessary computations, we incorporated a caching system for the Boltz-2 workers, ensuring that scores are computed only for molecules newly added to the Replay Buffer. This caching system also serves to trigger the termination of the generative screen. Once a substantial majority of sampled molecules have been previously observed, indicating policy convergence, the generation of new trajectories is halted. In our specific case, this condition was met after approximately 400,000 molecules were sampled (corresponding to roughly 16 hours of runtime), totaling nearly 1,000 GPU-hours of computation for the generative screen.

bioRxiv preprint doi: https://doi.org/10.1101/2025.06.14.659707; this version posted June 18, 2025. The copyright holder for this preprint (which was not certified by peer review) is the author/funder, who has granted bioRxiv a license to display the preprint in perpetuity. It is made available under aCC-BY 4.0 International license.

D Benchmarks and Baselines

D.1 Structure prediction

D.1.1 Benchmarks sets curation

We evaluate the models using recently released structures from the PDB, specifically from 01/01/24 to 12/31/2024. We filter the structures such that a target is kept if it has at least one monomer that is more than 40% sequence dissimilar to chains in the training data. For interfaces, we keep only interfaces where at least one of the two chains is sufficiently distinct, as defined above. In addition, we remove targets with resolution worse than 4.5A and with more than 1200 residues. This yields a final set of 2315 unique targets, from which we evaluate the subset of novel chains and interfaces.

D.1.2 Evaluation metrics

We evaluate the models using two sets of metrics:

- The lDDT (local Distance Difference Test), which we compute per modality (protein, RNA, DAN, ligands) as well as on specific interfaces of any two modalities.
- DockQ which we use to score antibody interfaces.

For each result, we provide the top-1 prediction across 5 samples according to the confidence model ranking.

For MD evaluation, we report the following metrics:

1. lDDT (local Distance Difference Test)
   - Precision: the average lDDT score from each predicted structure to its closest corresponding crystal structure.
   - Recall: the average lDDT score from each crystal structure to its closest predicted structure.
   - Diversity: the average structural dissimilarity between pairs of predicted structures, calculated as $1 - lDDT$.
2. RMSF-$C\alpha$ (root mean square fluctuation): measuring the atom-level flexibility over all conformations of the MD trajectory, and measuring its correlation to the generated data:
   - Spearman's rank correlation coefficient (ρ), pooled globally (by first aggregating all RMSF values across targets and then computing correlation metrics) as well as locally (by first computing the correlation within each target and then taking the median value across the dataset).
   - Pearson's correlation coefficient (r), also pooled globally and locally.
   - Root Mean Squared Error (RMSE) over the predicted and reference RMSF values, both locally and globally.

MD metrics were computed by taking 100 samples from Boltz-2 and other baseline models against 200 frames from the ATLAS and mdCATH trajectories.

D.1.3 Baselines

PDB baselines We evaluate Boltz-2 structural prediction results on the PDB against other state-of-the-art co-folding models, AlphaFold3, Chai-1, ProteinX, and Boltz-1. For all tools, we use the same inference parameters (5 recycling rounds, 5 samples, single seed) and the same MSA. We do not use templates during evaluation.

bioRxiv preprint doi: https://doi.org/10.1101/2025.06.14.659707; this version posted June 18, 2025. The copyright holder for this preprint (which was not certified by peer review) is the author/funder, who has granted bioRxiv a license to display the preprint in perpetuity. It is made available under aCC-BY 4.0 International license.

MD baselines To evaluate the ability of Boltz-2 to model multiple conformations, we compare against Boltz-1 [Wohlwend et al., 2025], AlphaFlow-MD base [Jing et al., 2024] and BioEmu with HPacker for side-chain reconstruction without MD relaxation [Lewis et al., 2025]. AlphaFlow is excluded from the ATLAS evaluation given that its training set largely overlaps with the test set constructed for Boltz-2. All models were run with the same MSA and sequence inputs.

D.2 Affinity prediction

This section will detail all the steps used to evaluate the affinity prediction. The evaluation addresses both hit discovery binary prediction, as well as the ranking of chemical series for hit-to-lead and lead optimization stages.

D.2.1 Benchmark sets curation

To rigorously evaluate the performance of our model across diverse binding tasks, we construct a curated suite of benchmark datasets targeting both continuous affinity value prediction and binary classification. These benchmarks are designed to reflect real-world drug discovery challenges, including hit-to-lead optimization and high-throughput screening.

Affinity value benchmarks. For the affinity regression task, we curate a validation set by selecting a diverse collection of hit-to-lead assays from our training corpus. The assays are chosen according to the following criteria:

- We retain only K_i measurements to ensure a higher degree of experimental consistency and lower measurement noise.
- Assays must exhibit a sufficiently large dynamic range in affinity values, enabling the model to distinguish strong from weak binders.
- We exclude assays with high correlation between affinity and molecular weight to minimize artifacts introduced by molecular series subselection.
- The selected assays span a diverse set of protein families to ensure broad generalization.

This filtering process yields 16 assays drawn from BindingDB and ChEMBL:

- ChEMBL assay_ids: 1528727, 438257, 1572912, 1705740, 157530, 454476, 2114176, 1527798, 769558.
- BindinDB DOIs: 10.7270/Q2JQ0ZNX, 10.7270/Q2ZC81K5, 10.7270/Q2VX0FFW, 10.7270/Q2RV0MMK, 10.7270/Q2VD71JR, 10.7270/Q26D5WBR, 10.7270/Q2VD72PZ.

We evaluate model performance on the following held-out affinity test sets:

- 2 subsets of the FEP+ benchmark: The OpenFE subset consisting of 876 protein–ligand complexes from hit-to-lead and lead optimization campaigns as well as a 4 targets subset (CDK2, TYK2, JNK1, P38; curated in the protein-ligand-benchmark [Hahn et al., 2022]) with 87 neutral compounds [Chen et al., 2023].
- A proprietary collection of internal hit-to-lead assays provided by Recursion.
- The CASP16 binding affinity challenge dataset.

Binary classification benchmarks. For binary binding prediction, we construct a validation set using six biochemical assays from the MF-PCBA dataset.

For the final test set, we select 10 biochemical high-throughput screening (HTS) assays from the MF-PCBA benchmark to maximize functional diversity. The following filtering steps are applied:

bioRxiv preprint doi: https://doi.org/10.1101/2025.06.14.659707; this version posted June 18, 2025. The copyright holder for this preprint (which was not certified by peer review) is the author/funder, who has granted bioRxiv a license to display the preprint in perpetuity. It is made available under aCC-BY 4.0 International license.

- We parse data directly from the MF-PCBA GitHub repository, adopting their assay-specific subselection strategy (e.g., retaining only ligands labeled as active in secondary confirmatory assays).
- Each assay is randomly downsampled to 50,000 protein–ligand complexes.
- We remove all compounds flagged as PAINS (Pan-Assay Interference Compounds) to mitigate false-positive artifacts.

The selected PubChem assay identifiers used for the test sets are: 743445, 485317, 2097, 493091, 2650, 485273, 504329, 489030, 588689, 588549.

Data leakage control. To avoid information leakage between training and evaluation splits, we apply strict sequence-level filtering. Specifically, we exclude from the training set any proteins with sequence similarity $\geq 90\%$ to proteins in the validation or test sets. This is implemented by first clustering all protein sequences in the affinity datasets using 'mmseqs easy-cluster ... –min-seq-id 0.9 –cov-mode 0 -c 0.01' [Hauser et al., 2016], and then removing any training protein that falls into a cluster shared with a validation or test protein. This filtering is applied to all benchmark datasets except for CASP16 and the Recursion internal assays: CASP16 data was released after our training data cutoff, and the Recursion benchmarks consist of proprietary internal targets not accessible to external sources. Moreover, for the FEP+ benchmark, we assess the impact of compound similarity in Figure D.2.1 by computing the maximum Tanimoto similarity of each test compound to the affinity value training set, followed by mean pooling across assays. We observe no significant dependence between prediction performance and compound similarity. We perform the same analysis on the CASP16 benchmark, obtaining maximum Tanimoto similarities of 0.41 for the L1000's compounds and 0.59 for the L3000's compounds, both sufficiently low to alleviate concerns about compound-level information leakage.

Benchmarking challenges. Improving benchmark design is essential for advancing affinity prediction across hit discovery, hit-to-lead, and lead optimization applications. Many widely used benchmarks employ subselection strategies that introduce artificial biases and obscure the challenges inherent in real-world campaigns. In binary activity datasets, noise from off-target effects and surrogate readouts further complicates learning and evaluation, often enabling models to exploit dataset artifacts rather than true structure–activity relationships. To assess model performance more meaningfully, we advocate for the creation of standardized benchmarks and careful curation to reduce experimental noise and spurious correlations, offering more reliable test sets for models intended to support early-stage compound prioritization and lead refinement.

D.2.2 Evaluation metrics

We evaluate our model on both continuous affinity prediction and binary binder classification tasks.

Affinity Value Prediction. All regression metrics are calculated per assay and averaged across assays with weight proportional to the number of compounds in each assay, ensuring that larger assays contribute proportionally to the overall performance summary. All predictions are converted to kcal/mol prior to computing the metrics. For the regression task, we report the following metrics:

1. Pearson's correlation coefficient (R), to measure linear correlation between predicted and true binding affinities.
2. Kendall's Tau (τ) rank correlation coefficient, to assess the monotonic agreement between predicted and experimental affinities within each assay.
3. Pairwise Mean Absolute Error (PMAE), calculated as the MAE over the pair-wise difference of affinity between any pair of compounds in a given assay.
4. Mean Absolute Error (MAE), computed between predicted and measured affinity values.

bioRxiv preprint doi: https://doi.org/10.1101/2025.06.14.659707; this version posted June 18, 2025. The copyright holder for this preprint (which was not certified by peer review) is the author/funder, who has granted bioRxiv a license to display the preprint in perpetuity. It is made available under aCC-BY 4.0 International license.

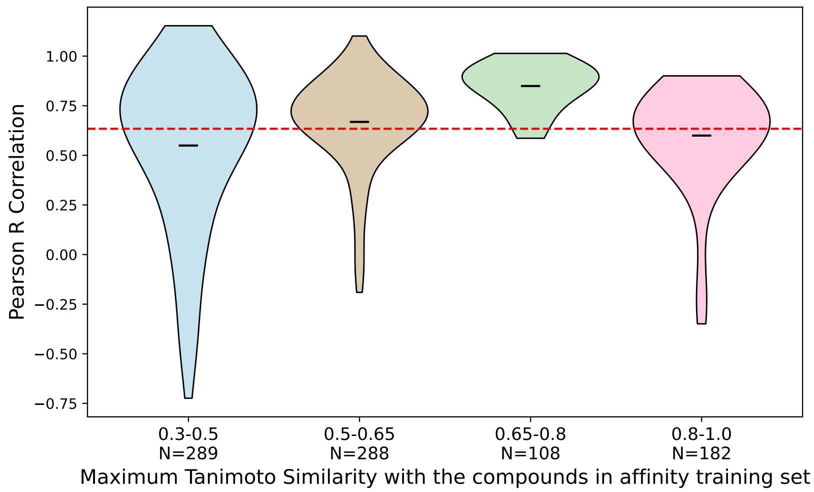

Figure 10: Relationship between compound similarity and prediction performance on the FEP+ benchmark. For each test compound, we compute the maximum Tanimoto similarity to any compound in the affinity training set. We then aggregate these values by computing the per-assay mean of the maximum similarity scores, and group the resulting assays into similarity bins: [0.3, 0.5], [0.5, 0.65], [0.65, 0.8], and [0.8, 1.0]. Each violin plot shows the distribution of per-assay Pearson correlation coefficients between predicted and ground truth affinities. The dashed red line indicates the weighted average of the per-assay Pearson correlations. We observe no strong dependence between compound similarity and predictive performance.

5. Percentage within 1 and 2 kcal/mol, indicating the fraction of predictions with absolute error below 1 and 2 kcal/mol, respectively.

For PMAE, MAE, and PW, we additionally report a *centered* version in which the predicted affinity values for each assay are translated to have the same mean as the corresponding ground truth values. This adjustment allows for a fair comparison with methods that only predict relative affinities, such as relative FEP.

Binary label Prediction. For the binary classification task, we compute the metrics per assay and average them uniformly across assays regardless of size. We report the following metrics:

1. Average Precision (AP), corresponding to the area under the Precision-Recall curve.
2. Enrichment Factor (EF) at top 0.5 %, 1%, 2% and 5% of the ranked list of compounds, reflecting early retrieval performance.
3. Area Under the Receiver Operating Characteristic Curve (AUROC), evaluating the model's ability to distinguish binders from non-binders.
4. *global* AUROC, where the metric is computed across the full set of compounds and targets jointly, rather than per-assay.

D.2.3 Baselines

Existing work for binding affinity prediction can be categorized into physics-based or ML-based approaches. Physics-based approaches include a range of methods across the cost-accuracy Pareto front, ranging from inexpensive Docking and QM-based scoring functions, through MD-based endstate approaches such as MM/PBSA to physically rigorous (alchemical) free energy simulations such as absolute and relative FEP. ML-based approaches can be roughly classified into structure-based and sequence-based methods. The former require the 3D (crystal) structure of the protein-ligand complex and are therefore usually only trained on the 20k complexes in PDBBind [Wang, 2024, Zhang et al., 2023]. The

bioRxiv preprint doi: https://doi.org/10.1101/2025.06.14.659707; this version posted June 18, 2025. The copyright holder for this preprint (which was not certified by peer review) is the author/funder, who has granted bioRxiv a license to display the preprint in perpetuity. It is made available under aCC-BY 4.0 International license.

latter only require knowledge about the compound SMILES and protein sequence and can therefore be trained on millions of experimental binding measurements without associated structures.

As an ML baseline, we select the sequence-based method *BACPI* [Li et al., 2022], given that the vast majority of our training data does not include structural information. BACPI consists of a 1D convolutional NN that processes the protein sequence and a graph attention transformer (GAT) that processes the ligand SMILES, connected by bi-directional attention. This baseline allows us to estimate the performance gain from leveraging predicted structural information compared to sequence-information only. In addition, we train ligand-only models to estimate the ligand bias in the data by deactivating the protein sequence CNN of BACPI (referred to as GAT in the following). We select the default hyperparameters of the original publication [Li et al., 2022], except for the batch size, which we increased to 32 to speed-up training on significantly larger datasets.

We also compare the performance of Boltz-2 to the following physical baselines: For ranking congeneric compounds in a hit-to-lead setting, we use our recently-published ABFE protocol [Wu et al., 2025] as well as the relative FEP protocols of OpenFE [Gowers et al., 2023] and FEP+ [Wang et al., 2015] for benchmarking. The results of FEP+ are intended to show the maximum attainable accuracy of current (commercial) FEP simulations by manually adjusting the protocol (input preparation, perturbation map, force field) to the system at hand after observing the error with respect to the experiment [Ross et al., 2023]. In addition, the results of the ABFE protocol [Wu et al., 2025] on the 4 target subset may also represent an optimistic estimate of its prospective performance given that the protocol was optimized based this dataset. In contrast, the OpenFE results are representative of automated open-source simulations (fixed protocol) [Horton, 2025]. We also compare to less expensive physics-based scoring functions based on docked poses obtained by Glide [Friesner et al., 2004] in the FEP+ dataset [Ross et al., 2023]. These include OpenEye's Chemgauss4 and an in-house DFTB3-based Fragment Molecular Orbital (FMO) code [Nishimoto and Fedorov, 2016, Guareschi et al., 2023]. As a representative of endstate approaches, we select the MM/PBSA implementation of AMBER [Miller III et al., 2012, Case et al., 2023]: Initial modeled complexes were thermalized with a position-restrained minimization and equilibration procedure, finishing with a 1 ns unrestrained simulation under NPT conditions. The final frame from this simulation was used for MM/PBSA scoring. In the binary hit discovery setting, we select the commercial Docking engine OpenEye FRED [McGann, 2011] to predict binding poses. Any undefined R/S or E/Z stereocenters in input molecules were exhaustively enumerated and molecular conformers for FRED docking were generated with OpenEye's OMEGA with default sampling options. We rank compounds by highest Chemgauss4 efficiency: Chemgauss4 score squared, divided by the number of heavy atoms. In the case of multiple stereoisomers per input molecule, we select the isomer with the highest score. In the absense of experimental crystal structures in MF-PCBA, we co-fold the median-weight active compound of each assay with Boltz-2 to obtain the receptor structure for Docking. In all cases, we prepare protein structures with OpenEye Spruce.

bioRxiv preprint doi: https://doi.org/10.1101/2025.06.14.659707; this version posted June 18, 2025. The copyright holder for this preprint (which was not certified by peer review) is the author/funder, who has granted bioRxiv a license to display the preprint in perpetuity. It is made available under aCC-BY 4.0 International license.

E Extended Results

In this section, we present additional results, evaluation metrics, and analyses covering the various components of the model. Due to the scale of Boltz-2, comprehensive ablation studies isolating the impact of each architectural or training component on final performance are not computationally feasible.

Note that this paper is still a preprint in preparation. In the coming weeks, we will integrate even more results to the paper including evaluations of the template, contact and pocket conditioning as well as more challenging prospective evaluations of our small-molecule design pipelines.

E.1 Molecular Dynamics

Tables 9 and 10 present the precise measurements of the RMSF correlations and RMSE of the various models on a per-target and global basis for holdout MD datasets. Figure 11 displayes the global correlations between predicted and groundtruth RMSF values.

Metric	Boltz-2 - Xray	Boltz-2 - MD	Boltz-1	AlphaFlow	BioEmu
↑ Global RMSF r	0.48	**0.67**	0.46	0.24	0.53
↑ Per-target RMSF r	0.72	**0.79**	0.70	0.77	0.77
↑ Global RMSF ρ	0.61	**0.65**	0.52	0.45	0.44
↑ Per-target RMSF ρ	0.78	**0.81**	0.76	0.76	0.78
↓ Global RMSF RMSE	192	**157**	197	229	212
↓ Per-target RMSF RMSE	21.71	16.30	22.92	18.74	**14.85**

Table 9: mdCATH test set. Comparison of methods based on RMSF metrics: correlation (r), Spearman's rank correlation (ρ), and mean squared error (MSE), both globally and per target. Boltz-2 is run with MD and X-ray method conditioning.

Metric	Boltz-2 - Xray	Boltz-2 - MD	Boltz-1	BioEmu
↑ Global RMSF r	0.57	**0.65**	0.38	0.56
↑ Per-target RMSF r	0.76	**0.85**	0.77	0.83
↑ Global RMSF ρ	0.63	**0.76**	0.67	0.63
↑ Per-target RMSF ρ	0.82	**0.87**	0.83	0.81
↓ Global RMSF RMSE	185	**155**	218	209
↓ Per-target RMSF RMSE	17.42	**12.35**	19.62	15.04

Table 10: ATLAS test set. Comparison of methods based on RMSF metrics: correlation (r), Spearman's rank correlation (ρ), and mean squared error (MSE), both globally and per target. Boltz-2 is run with MD and X-ray method conditioning.

bioRxiv preprint doi: https://doi.org/10.1101/2025.06.14.659707; this version posted June 18, 2025. The copyright holder for this preprint (which was not certified by peer review) is the author/funder, who has granted bioRxiv a license to display the preprint in perpetuity. It is made available under aCC-BY 4.0 International license.

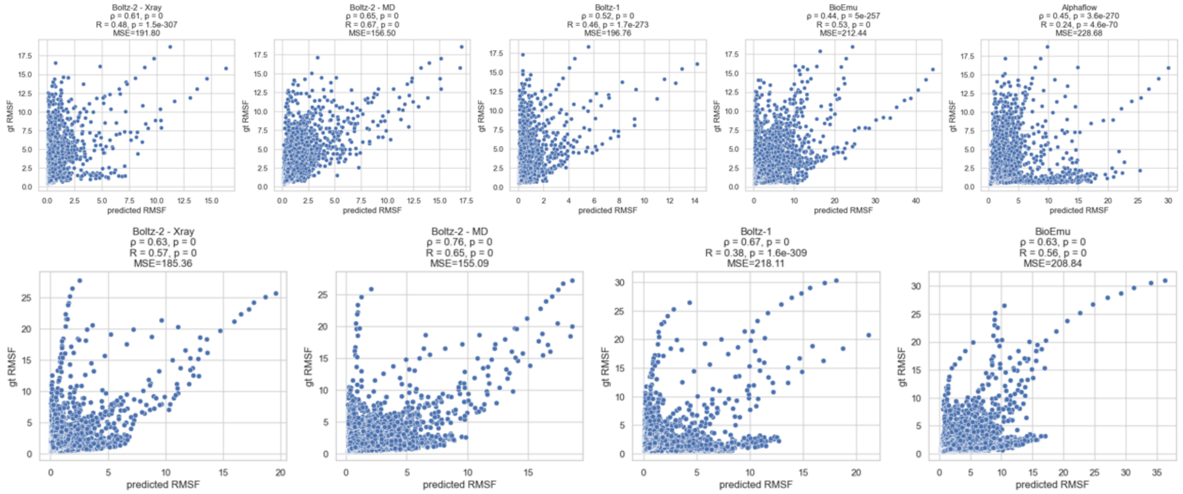

Figure 11: Global RMSF Spearman, Pearson and MSE metrics for the mdCATH (top) and ATLAS (bottom) holdout sets.

bioRxiv preprint doi: https://doi.org/10.1101/2025.06.14.659707; this version posted June 18, 2025. The copyright holder for this preprint (which was not certified by peer review) is the author/funder, who has granted bioRxiv a license to display the preprint in perpetuity. It is made available under aCC-BY 4.0 International license.

E.2 Affinity prediction

E.2.1 Public benchmarks

In this section, we provide the complete set of quantitative results for the model's performance across all public benchmarks used in our evaluation. These are intended to supplement the main text by offering a more detailed view of the metrics and trends observed in each dataset.

Tables 11 and 12 present the comprehensive benchmark results for the FEP+ dataset, including both OpenFE subset and the focused 4-target evaluation. Table 15 shows the model's performance on our affinity values validation set, which was constructed to span a representative set of high-quality hit-to-lead assays. In Figure 12, we visualize the predicted versus measured affinity values for each assay in the validation set. Each scatter plot is annotated by protein class (e.g., kinases, GPCRs, proteases), enabling visual assessment of potential systematic trends. While we do not observe any systematic degradation or improvement in performance attributable to specific protein classes, we do find substantial variability across assays, with Pearson correlations ranging from 0.732 to 0.056. Table 14 reports Boltz-2's results on the CASP16 blind challenge, alongside the ML baselines and the top-6 ranked entries from the competition. Lastly, Table 13 presents the complete evaluation metrics for the MF-PCBA test set, while Figure 13 complements this by showing ROC curves and activity histograms for individual assays.

Table 11: OpenFE subset of the FEP+ benchmark. Comparison of Boltz-2, ML baselines and free energy perturbation.

method	type	Pearson R	Kendall tau	PMAE	MAE		Perc. within 1		Perc. within 2	
		target avg.	target avg.	target avg.	non-cent.	cent.	non-cent.	cent.	non-cent.	cent.
**Boltz-2**	ML	0.62	0.46	0.93	1.22	0.64	0.49	0.80	0.82	0.96
Boltz-2 iptm	ML	-0.07	-0.05	N/A	N/A	N/A	N/A	N/A	N/A	N/A
GAT	ML	0.28	0.20	1.30	1.42	0.91	0.40	0.64	0.75	0.92
BACPI	ML	0.29	0.19	1.21	1.44	0.85	0.40	0.67	0.74	0.94
OpenFE	physics	0.63	0.47	1.37	N/A	0.94	N/A	0.65	N/A	0.91
FEP+	physics	0.72	0.53	0.94	N/A	0.64	N/A	0.79	N/A	0.97

Table 12: 4 target subset of the FEP+ benchmark. Comparison of Boltz-2, ML baselines and an extensive set of physics-based methods.

method	type	time	Pearson R	Kendall tau	PMAE	MAE		Perc. within 1		Perc. within 2	
			target avg.	target avg.	target avg.	non-cent.	cent.	non-cent.	cent.	non-cent.	cent.
**Boltz-2**	ML	20 GPU sec	0.66	0.48	0.85	0.75	0.59	0.69	0.83	0.97	0.98
Boltz-2 iptm	ML	5 GPU sec	0.04	0.09	N/A	N/A	N/A	N/A	N/A	N/A	N/A
BACPI	ML	0.48 GPU ms	0.14	0.09	1.18	1.40	0.82	0.43	0.62	0.73	1.00
GAT	ML	0.18 GPU ms	0.40	0.28	1.07	1.19	0.71	0.43	0.72	0.86	0.95
OpenFE	physics	6-12 GPU hours	0.66	0.51	1.09	N/A	0.75	N/A	0.70	N/A	0.98
FEP+	physics	-	0.78	0.63	0.77	N/A	0.53	N/A	0.85	N/A	1.00
ABFE	physics	>20 GPU hours	0.75	0.54	0.95	2.47	0.65	0.11	0.79	0.40	0.98
FMO	physics	2-10 CPU min	0.55	0.38	N/A	N/A	N/A	N/A	N/A	N/A	N/A
MM/PBSA	physics	10-15 CPU min	0.18	0.16	N/A	N/A	N/A	N/A	N/A	N/A	N/A
Chemgauss4	physics	20-30 CPU sec	0.26	0.17	N/A	N/A	N/A	N/A	N/A	N/A	N/A

Table 13: MF-PCBA test set. Comparison of Boltz-2 with ML baselines, confidence score and Chemgauss4 Docking score.

method	AP	EF at 0.5%	EF at 1%	EF at 2%	EF at 5%	AUROC	
	target avg.	target avg.	target avg.	target avg.	target avg.	target avg.	global
**Boltz-2**	0.0248	18.3916	13.9540	10.5706	7.0448	0.8122	0.8056
Chemgauss4	0.0051	1.9969	2.2257	2.1136	1.6462	0.5450	0.5706
Boltz-2 iptm	0.0046	2.4242	3.1728	2.6881	2.2263	0.5657	0.6134
GAT	0.0133	11.1179	8.9897	7.5630	5.9055	0.7928	0.7867
BACPI	0.0131	9.4818	9.2397	7.3983	5.5533	0.7575	0.7205

bioRxiv preprint doi: https://doi.org/10.1101/2025.06.14.659707; this version posted June 18, 2025. The copyright holder for this preprint (which was not certified by peer review) is the author/funder, who has granted bioRxiv a license to display the preprint in perpetuity. It is made available under aCC-BY 4.0 International license.

Table 14: CASP16 competition. Comparison of Boltz-2 and the ML baselines with the top-6 highest ranked participants.

method	Pearson R target avg.	Kendall tau target avg.	Pairwise MAE target avg.	MAE non-cent.	MAE cent.	Perc. within 1 non-cent.	Perc. within 1 cent.	Perc. within 2 non-cent.	Perc. within 2 cent.
**Boltz-2**	0.65	0.45	1.36	1.28	0.95	0.48	0.61	0.81	0.90
LG016	0.54	0.42	1.43	1.09	1.03	0.53	0.51	0.83	0.91
LG082	0.38	0.36	1.55	1.17	1.12	0.49	0.47	0.83	0.85
LG204	-	0.34	-	-	-	-	-	-	-
LG055	0.47	0.33	1.59	1.29	1.12	0.45	0.53	0.78	0.84
LG207	0.38	0.32	1.65	1.32	1.19	0.48	0.47	0.74	0.83
LG008	0.38	0.29	1.62	1.43	1.16	0.39	0.51	0.72	0.85
Boltz-2 iptm	0.12	0.07	N/A	N/A	N/A	N/A	N/A	N/A	N/A
GAT	0.50	0.35	1.58	1.28	1.13	0.44	0.49	0.79	0.84
BACPI	0.41	0.31	1.55	1.25	1.10	0.45	0.51	0.81	0.89

Table 15: Hit-to-lead affinity validation set. Comparison between Boltz-2 and ML baselines.

method	Pearson R target avg.	Kendall tau target avg.	PMAE target avg.	MAE non-cent.	MAE cent.	Perc. within 1 non-cent.	Perc. within 1 cent.	Perc. within 2 non-cent.	Perc. within 2 cent.
**Boltz-2**	0.4246	0.2855	1.2046	1.7001	0.8569	0.3071	0.6555	0.6223	0.9351
GAT	0.2512	0.1795	1.3117	1.4261	0.9308	0.4288	0.6231	0.7433	0.9043
BACPI	0.1997	0.1330	1.2855	1.8775	0.9103	0.2913	0.6260	0.5861	0.9120

E.2.2 Private benchmarks

This section supplements the main text discussion about the benchmarking of Boltz-2 on blinded, past experimental binding assays performed at Recursion, which represents a more rigorous evaluation of the types of problems we expect Boltz-2 to be exposed to in real-world drug discovery projects. Each assay has hundreds of compounds that were screened during the hit-to-lead stage. Unlike the deep learning baselines, Boltz-2 still achieves decent correlation with the experiment on average (table E.2.2). As displayed in figure 14, the model achieves respectable performance on these assays, achieving an average Pearson R = 0.39, only slightly worse than in the validation set (R = 0.42). However, the centered MAE = 1.36kcal/mol is significantly worse compared to the validation set (MAE = 0.86kcal/mol). In addition, the performance varies noticably between targets, ranging from Person R = 0.165 to R = 0.634 and centered MAE from MAE = 0.855kcal/mol to MAE = 1.734kcal/mol, suggesting that performance in practice will strongly depend on the project at hand. These results highlight challenges in real-world drug discovery projects that may be insufficiently reflected in public benchmarks. This effect has been recently observed for benchmarking OpenFE as well, where performance significantly dropped on private data compared to the FEP+ benchmark [Horton, 2025].

Table 16: Benchmark on 8 blinded, private hit-to-lead binding assays. Comparison of Boltz-2 with ML baselines.

method	Pearson R target avg.	Kendall tau target avg.	Pairwise MAE target avg.	MAE non-cent.	MAE cent.	Perc. within 1 non-cent.	Perc. within 1 cent.	Perc. within 2 non-cent.	Perc. within 2 cent.
**Boltz-2**	0.39	0.23	1.91	1.67	1.36	0.38	0.45	0.66	0.77
GAT	0.16	0.11	2.16	1.84	1.47	0.32	0.41	0.61	0.73
BACPI	0.11	0.06	2.11	1.87	1.44	0.32	0.42	0.59	0.74

bioRxiv preprint doi: https://doi.org/10.1101/2025.06.14.659707; this version posted June 18, 2025. The copyright holder for this preprint (which was not certified by peer review) is the author/funder, who has granted bioRxiv a license to display the preprint in perpetuity. It is made available under aCC-BY 4.0 International license.

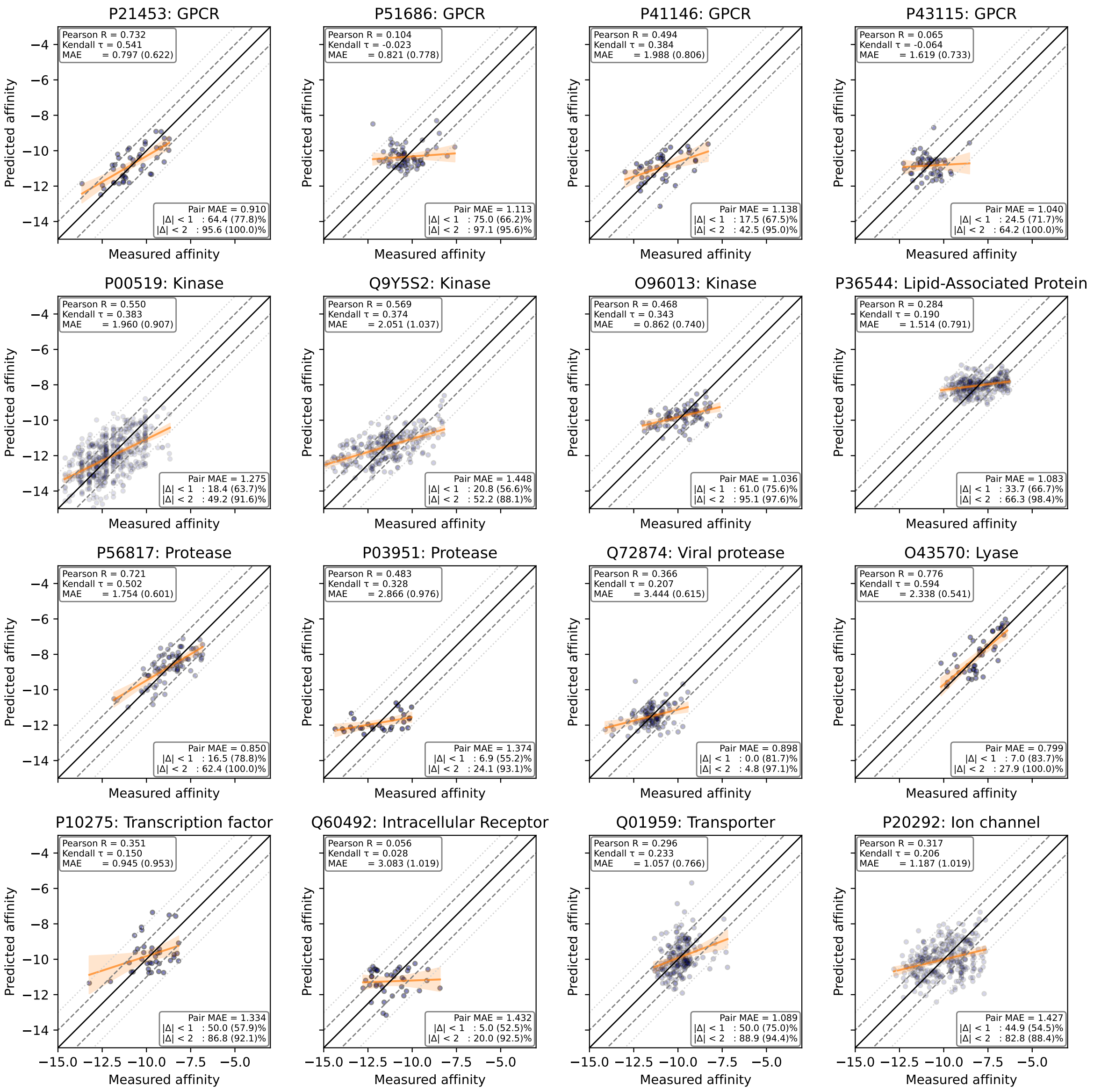

Figure 12: Assay in the validation set, in kcal/mol. The grey lines represent an absolute error of 1 and 2 kcal/mol. $|\Delta| < \epsilon$ represents the percentage of the data that falls within an absolute error of ϵ. The number in parenthesis represent the centered metrics.

bioRxiv preprint doi: https://doi.org/10.1101/2025.06.14.659707; this version posted June 18, 2025. The copyright holder for this preprint (which was not certified by peer review) is the author/funder, who has granted bioRxiv a license to display the preprint in perpetuity. It is made available under aCC-BY 4.0 International license.

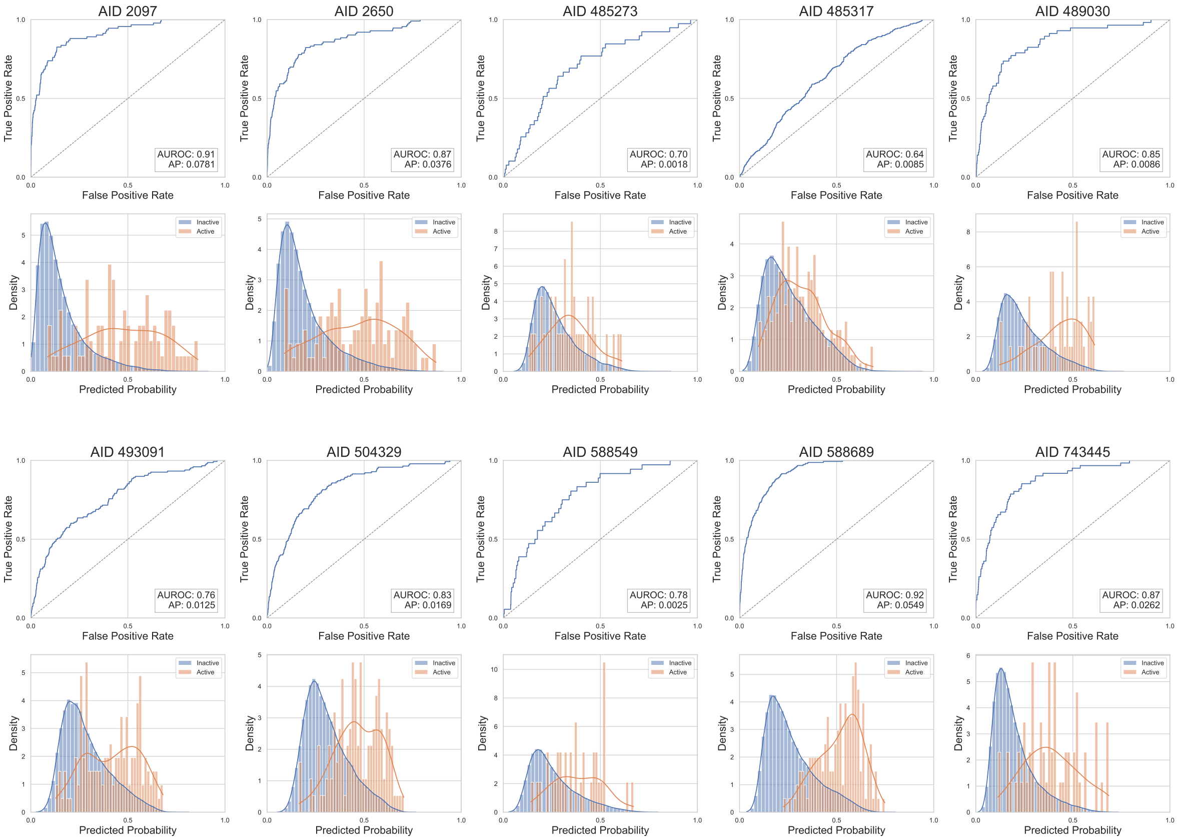

Figure 13: Receiver operating characteristic curves and histograms of binder and decoy distributions for the assays in the MF-PCBA test set.

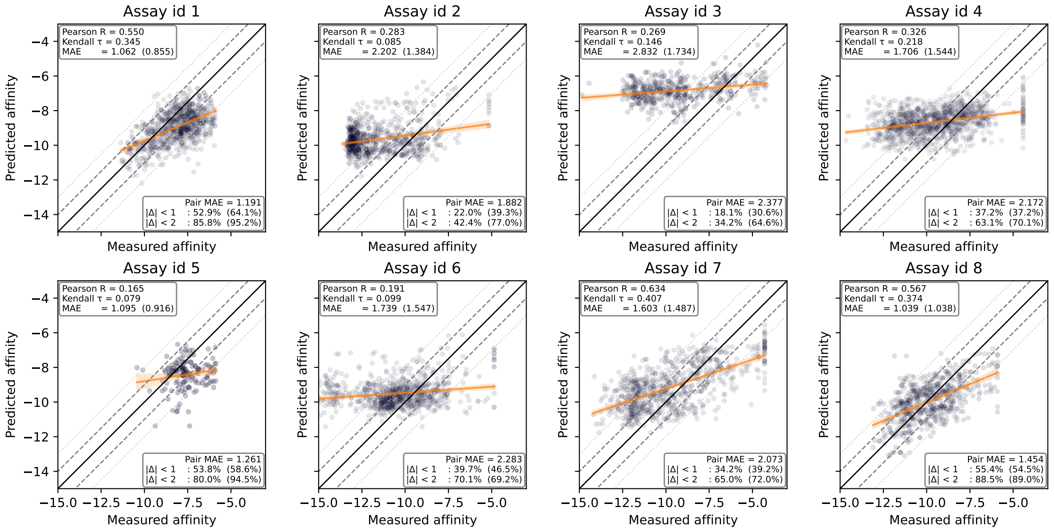

Figure 14: Boltz-2 predictions on 8 blinded targets from private assay, in kcal/mol. The grey lines represent an absolute error of 1 and 2 kcal/mol. $|\Delta| < \epsilon$ represents the percentage of the data that falls within an absolute error of ϵ. The number in parenthesis represent the centered metrics.

bioRxiv preprint doi: https://doi.org/10.1101/2025.06.14.659707; this version posted June 18, 2025. The copyright holder for this preprint (which was not certified by peer review) is the author/funder, who has granted bioRxiv a license to display the preprint in perpetuity. It is made available under aCC-BY 4.0 International license.

E.3 Prospective virtual screens

In this section, we provide addtionnal details and results from the filtering pipeline of the prospective virtual screens of Section 5.4, visualisations of all of the ABFE-screened molecules from Figure 8, 3D renderings of the co-folded structure of the top two ligands (as ranked by ABFE) with the TYK2 protein, and a similarity analysis with the TYK2 ligands that were contained in Boltz-2 structural training data through the PDB.

E.3.1 Ligand filtering pipeline

Table E.3.1 shows the number of remaining compounds after each filtering stage of our screening pipeline, for both our generative SynFlowNet screen and the fixed virtual screens of Enamine's HLL and Kinase libraries. The first and most important stage is computing the scores using Boltz-2 for each of the compounds under consideration. For SynFlowNet, 117, 199 unique compounds were scored out of the $400k$ samples from the model throughout the training of the model. We sequentially (1) removed all compounds with a score below 0.5, (2) discarded all the compounds not contained in the REAL space, to guarantee purchasability, (3) performed multi-parameter optimization (MPO) the scores computed by both ensemble models to be above 0.9, and (4) enumerated all undefined R/S or E/Z stereocenters of the selected molecules and removed compounds with 4 or more stereoisomers to reduce the ABFE simulation cost. To finalise the candidate set for ABFE-validation, we selected 10 diverse compounds among the 93 remaining candidates using Tanimoto fingerprint similarity. For fixed libraries (HLL and Kinase), we notice that since these libraries were not assembled specifically to optimise against our target, contrary to the SynFlowNet stream, we are left with only a few hundreds remaining compounds after imposing a score threshold of 0.5. The compounds being already purchasable, we do not test containment to the REAL space and simply select the set of 10 compounds that provide the best joint scores across the ensemble. At this point, not enough samples were left to further maximise diversity. The results in Figure 15 show how the score distributions of the selected compounds differ from a random control set and a set of public TYK2 binders from the protein-ligand benchmark Hahn et al. [2022].

Table 17: Number of compounds after each filtering stage of the screening pipeline for all five streams.

Stream	Initial		Score > 0.5		in REAL		MPO 1&2		Max Diversity		Final
SynflowNet screen	117,199	→	16,317	→	1,996	→	93	→	10	→	10
Enamine HLL screen	460,160	→	239	→	–	→	10	→	–	→	10
Enamine Kinase Lib screen	64,960	→	506	→	–	→	10	→	–	→	10
Random REAL sample	1,000	→	–	→	1,000	→	–	→	10	→	10
Public TYK2 binders	10	→	–	→	–	→	–	→	–	→	10

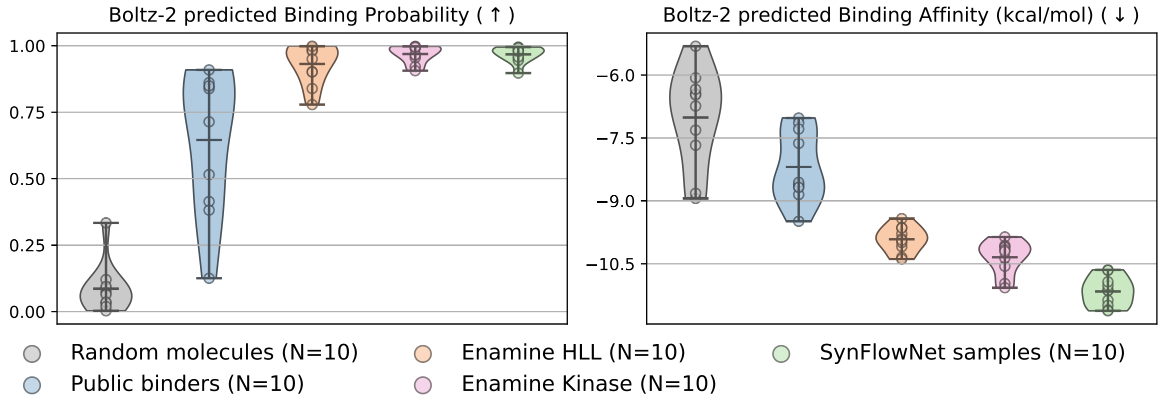

Figure 15: In virtual screening, Boltz-2 identifies what it believes to be high-scoring compounds, with the SynFlowNet obtaining higher scores on average. These potential binders are then tested with ABFE (results in Figure 8).

bioRxiv preprint doi: https://doi.org/10.1101/2025.06.14.659707; this version posted June 18, 2025. The copyright holder for this preprint (which was not certified by peer review) is the author/funder, who has granted bioRxiv a license to display the preprint in perpetuity. It is made available under aCC-BY 4.0 International license.

E.3.2 ABFE validation protocol

Before ABFE evaluation, we select the most dominant tautomer state at pH=7.4 using ChemAxon. Subsequently, we use Boltz-2 to co-fold the selected compounds with TYK2 and call our Boltz-ABFE protocol to estimate ABFE values. For compounds with multiple stereoisomers, we select the isomer with the larger ABFE value.

E.3.3 Ligands visualisations

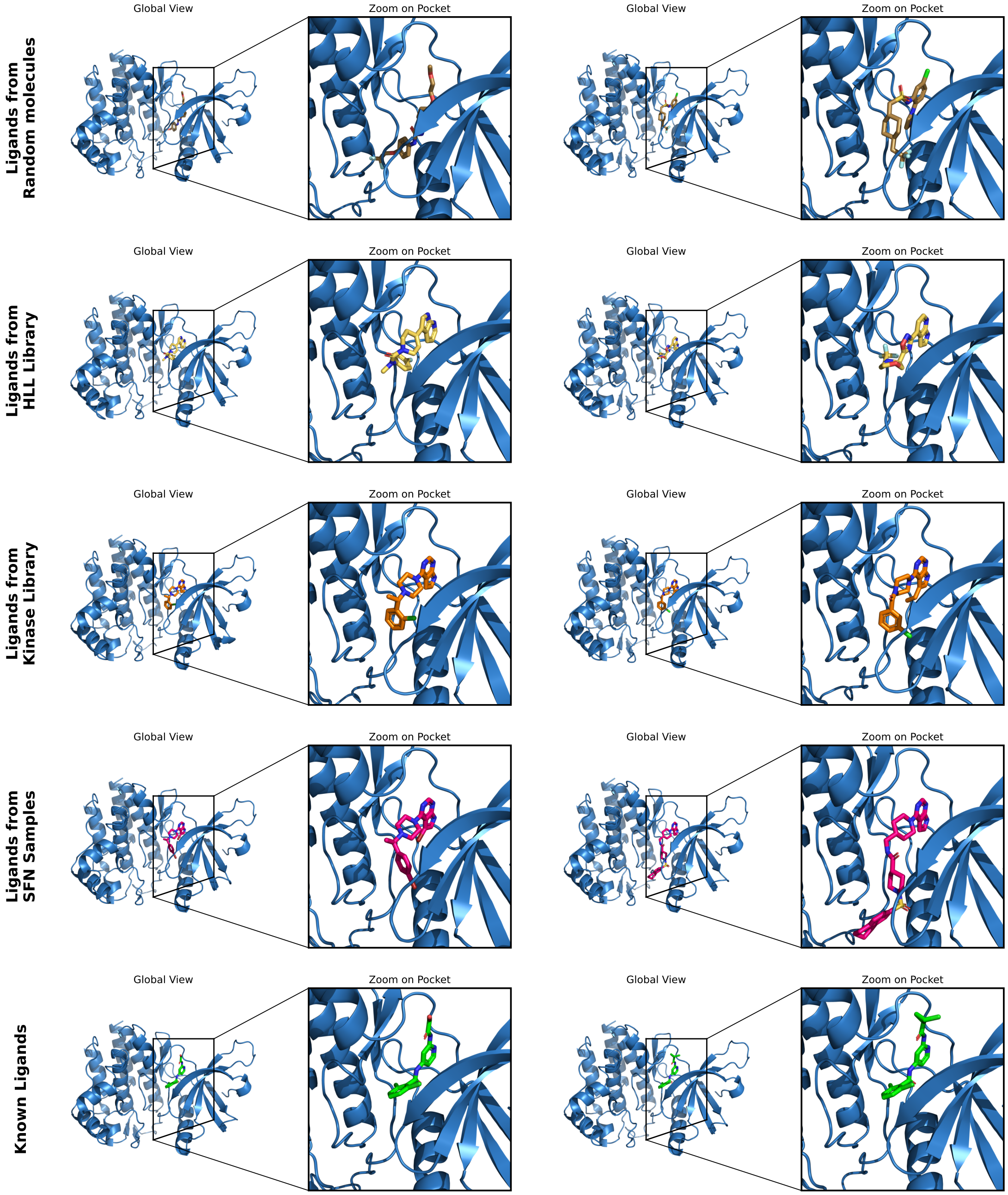

Figure 16: Visualization of the binding pose of the top two ligands found by each virtual screening method, as ranked by ABFE with the TYK2 protein in blue. *Note: We remind the reader that the molecules were solely optimized for their Boltz-2 score. Other properties, such as toxicity, solubility, metabolism, etc. are ignored.*

bioRxiv preprint doi: https://doi.org/10.1101/2025.06.14.659707; this version posted June 18, 2025. The copyright holder for this preprint (which was not certified by peer review) is the author/funder, who has granted bioRxiv a license to display the preprint in perpetuity. It is made available under aCC-BY 4.0 International license.

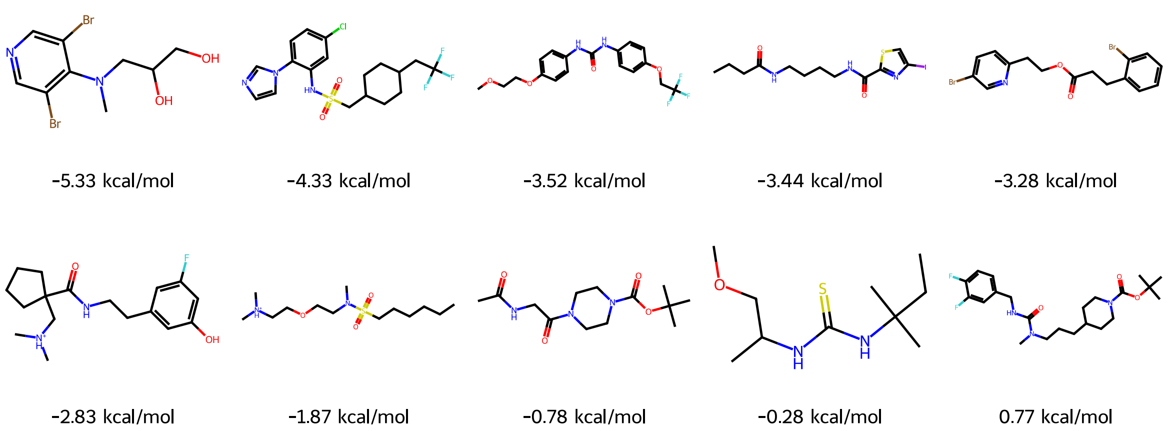

Figure 17: Random molecules selected for ABFE evaluation.

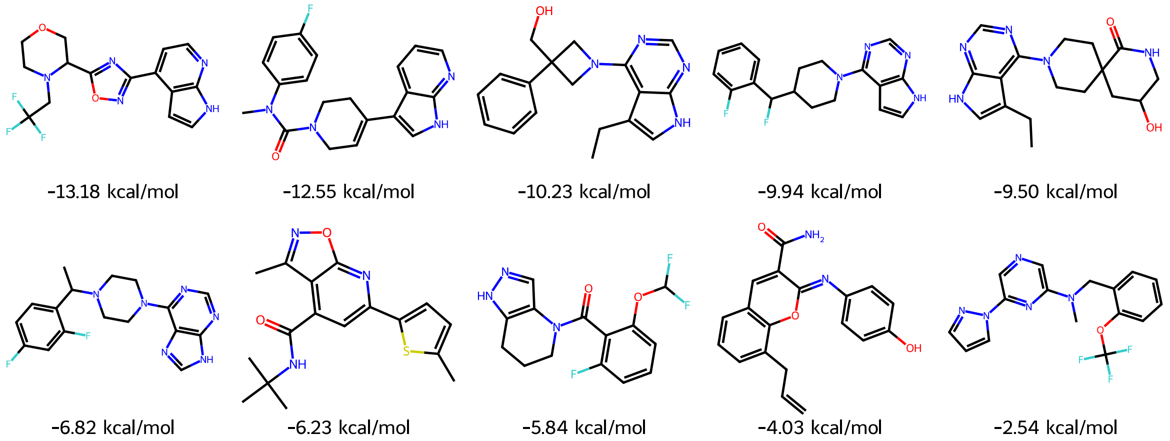

Figure 18: Molecules from the HLL library selected for ABFE evaluation.

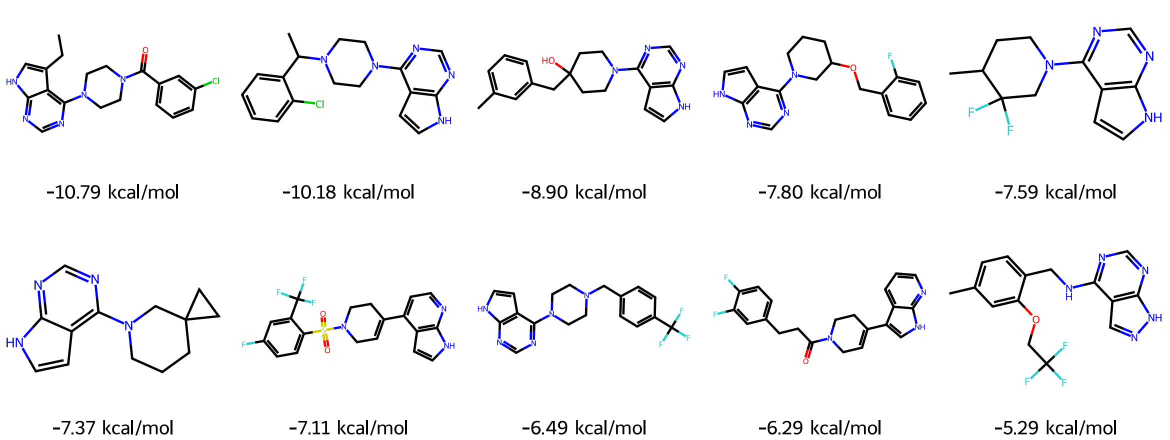

Figure 19: Molecules from the Kinase library selected for ABFE evaluation.

bioRxiv preprint doi: https://doi.org/10.1101/2025.06.14.659707; this version posted June 18, 2025. The copyright holder for this preprint (which was not certified by peer review) is the author/funder, who has granted bioRxiv a license to display the preprint in perpetuity. It is made available under aCC-BY 4.0 International license.

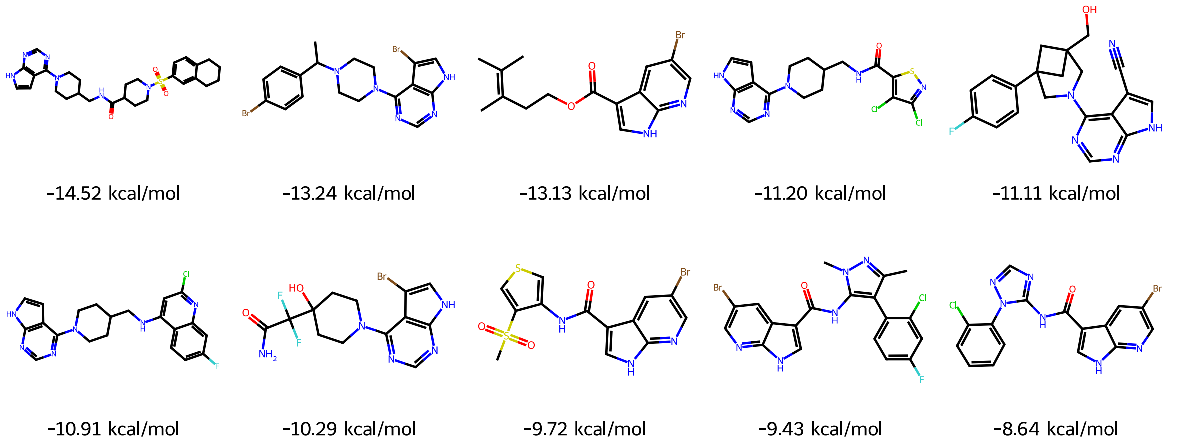

Figure 20: Molecules generated by SynFlowNet for ABFE evaluation. *Note: We remind the reader that the molecules were solely optimized for their Boltz-2 score. All other properties, such as toxicity, solubility, metabolism, etc. are ignored.*

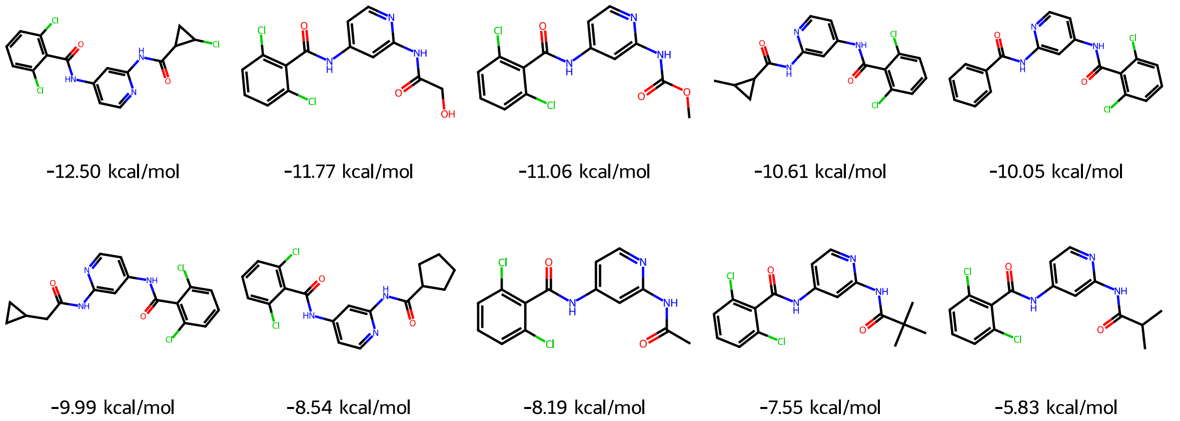

Figure 21: Public TYK2 binders from the protein-ligand benchmark selected for ABFE evaluation.

E.3.4 Similarity analysis to public TYK2-binders

The Boltz-2 structure module was trained using TYK2 protein-ligand complexes from the PDB. To assess whether Boltz-2 successfully generalized within the ligand space to steer the generation of novel candidate TYK2 binders, we collected the 47 public TYK2 inhibitors from the KLIFS database [Kanev et al., 2021] that correspond to the co-crystalized TYK2 inhibitors from the PDB. Then, we computed their Morgan fingerprint Tanimoto similarity to the ABFE-tested compounds generated by SynFlowNet. The similarity scores were computed both on the Murcko scaffolds and for the full molecules for each compounds pair (in Figure 22 we show the similarity matrix between scaffold pairs since this is the most restrictive metric). As shown in Figure 22, none of the SynFlowNet–KLIFS ligand pairs exhibited high similarity, with a maximum Tanimoto score of just 0.396 between the most similar scaffold pairs. In Figure 23, we show the most similar KLIFS ligand for every ABFE-tested ligand generated by SynFlowNet. We observe that the model captured the hinge-binding relevance of a pyrrolopyrimidine-like heterocycle, a well-established motif in orthosteric kinase inhibitor design. However, it reuses this scaffold across a range of diverse chemotypes that remain structurally distinct

bioRxiv preprint doi: https://doi.org/10.1101/2025.06.14.659707; this version posted June 18, 2025. The copyright holder for this preprint (which was not certified by peer review) is the author/funder, who has granted bioRxiv a license to display the preprint in perpetuity. It is made available under aCC-BY 4.0 International license.

from their closest KLIFS counterparts.

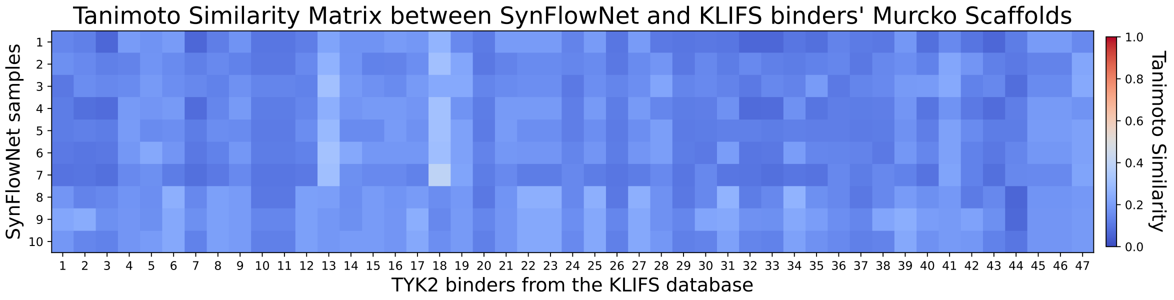

Figure 22: Similarity matrix between the 10 generated compounds from the SynFlowNet screen, and the TYK2 binders from the PDB as obtained from the KLIFS database. The generated compounds show significant novelty, with at most 0.396 Tanimoto similarity with known binders.

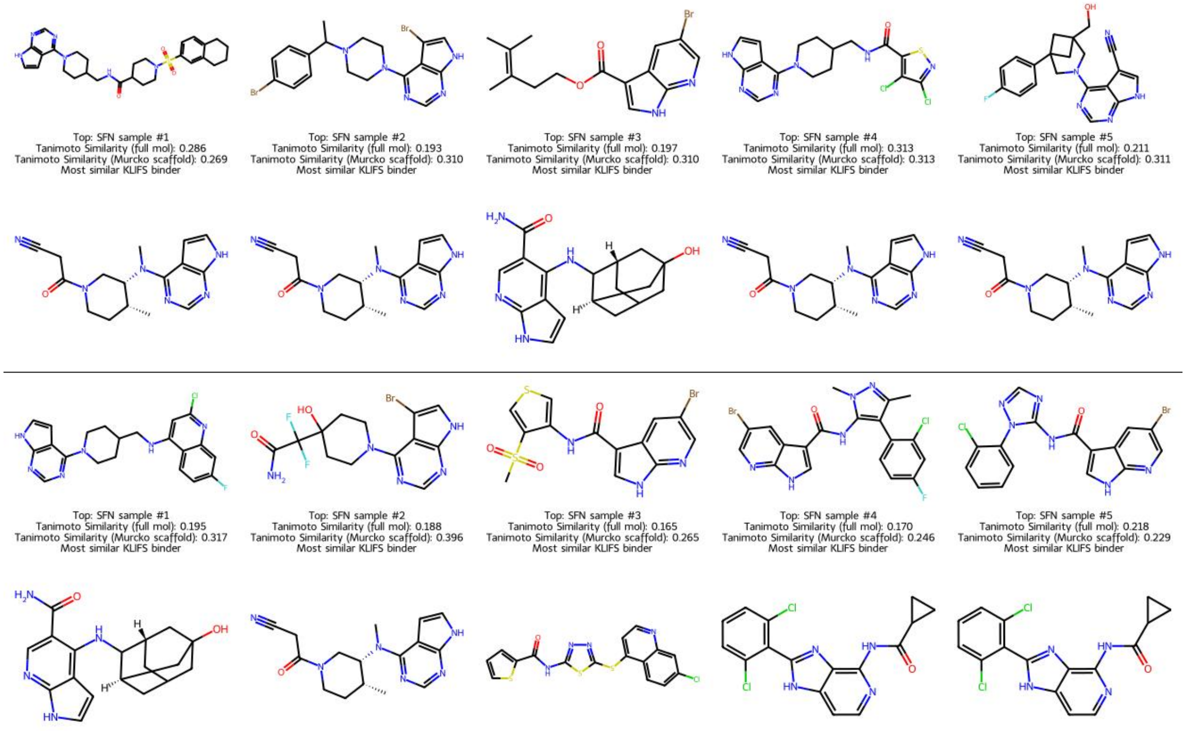

Figure 23: For each generated ligand from our SynFlowNet model, we show the most similar TYK2 binder from the PDB as curated in the KLIFS database. Although we find some similar structural groups, the generated ligands exhibit significant novelty throughout their entire structures, encompassing both the scaffolds and their decorations. *Note: We remind the reader that the molecules were solely optimized for their Boltz-2 score. Other properties, such as toxicity, solubility, metabolism, etc. are ignored.*
